# Small molecule inhibitors of transcriptional cyclin-dependent kinases impose HIV-1 latency, presenting “block and lock” treatment strategies

**DOI:** 10.1128/aac.01072-23

**Published:** 2024-02-06

**Authors:** Riley M. Horvath, Zabrina L. Brumme, Ivan Sadowski

**Affiliations:** 1 Department of Biochemistry and Molecular Biology Molecular Epigenetics Group, LSI, University of British Columbia, Vancouver, British Columbia, Canada; 2 Faculty of Health Sciences, Simon Fraser University, Burnaby, British Columbia, Canada; 3 British Columbia Centre for Excellence in HIV/AIDS, Vancouver, British Columbia, Canada; IrsiCaixa Institut de Recerca de la Sida, Barcelona, Spain

**Keywords:** CDK7, CDK8, CDK9, CDK19, YKL-5-124, LDC000067, Senexin A, HIV-1, latency, transcription, TFIIH, mediator kinase, P-TEFb, block and lock

## Abstract

Current antiretroviral therapy for HIV-1 infection does not represent a cure for infection as viral rebound inevitably occurs following discontinuation of treatment. The “block and lock” therapeutic strategy is intended to enforce proviral latency and durably suppress viremic reemergence in the absence of other intervention. The transcription-associated cyclin-dependent protein kinases (tCDKs) are required for expression from the 5´ HIV-1 long-terminal repeat, but the therapeutic potential of inhibiting these kinases for enforcing HIV-1 latency has not been characterized. Here, we expanded previous observations to directly compare the effect of highly selective small molecule inhibitors of CDK7 (YKL-5-124), CDK9 (LDC000067), and CDK8/19 (Senexin A), and found each of these prevented HIV-1 provirus expression at concentrations that did not cause cell toxicity. Inhibition of CDK7 caused cell cycle arrest, whereas CDK9 and CDK8/19 inhibitors did not, and could be continuously administered to establish proviral latency. Upon discontinuation of drug administration, HIV immediately rebounded in cells that had been treated with the CDK9 inhibitor, while proviral latency persisted for several days in cells that had been treated with CDK8/19 inhibitors. These results identify the mediator kinases CDK8/CDK19 as potential “block and lock” targets for therapeutic suppression of HIV-1 provirus expression.

## INTRODUCTION

The barrier toward a cure for HIV-1 is the population of extremely long-lived CD4^+^ T cells that harbor latent provirus. Antiretroviral therapy (ART) is capable of halting disease progression but has no effect on the latent HIV-1 reservoir and, consequently, viral replication rapidly rebounds upon discontinuation of treatment (1, 2). The seemingly antithetical proposed “shock and kill” and “block and lock” therapeutic strategies are intended to eliminate the requirement of lifelong ART through manipulation of latent provirus expression (3). The “shock and kill” strategy would involve pharmacological reactivation by latency reversal agents (LRAs) and encouraging destruction of infected cells through viral cytotoxicity and immunological surveillance (4, 5). Development of this strategy faces formidable barriers as it may be impossible to reactivate the majority of latent provirus (6–8) and HIV-1-specific CD8^+^ lymphocyte-mediated clearance becomes inefficient in individuals on ART (9, 10). Given these limitations, the “block and lock” strategy, by which durable latency is enforced using latency promoting agents (LPAs) to suppress viral rebound in the absence of ART, might represent a more feasible therapeutic option (11, 12). Significant recent attention has been focused on the development of these strategies to modulate expression of HIV-1 provirus, and there is the potential that sequential treatment with LRAs and LPAs may contribute to an effective strategy to promote remission. For example, LRAs in combination with other intervention might be first applied to promote killing of as many latently infected cells as possible, which could then be followed with a latency promoting agent to silence the remaining provirus (13).

Of the many factors which regulate expression of HIV-1 provirus, the transcription-associated cyclin-dependent kinases (tCDKs) represent important potential targets for LPAs. The RNA polymerase II-associated protein kinases, including CDK7, CDK8, CDK9, and CDK19, are regulated by association with cyclin proteins whose abundance, unlike cyclins that control CDKs that regulate cell cycle progression, does not fluctuate throughout the cell cycle (14), but rather function to regulate specific activities of RNA polymerase II (15). Each of the tCDKs plays significant roles for regulation of HIV-1 provirus expression. Phosphorylation of serine 5 on the C-terminal domain (CTD) of the largest RNA polymerase II subunit by CDK7, as part of general transcription factor IIH (TFIIH) (16, 17), is required for clearance of RNA polymerase II (RNAPII) from the core LTR promoter (18), and recruitment of TFIIH to the HIV-1 LTR was shown to be a limiting step for reactivation of HIV-1 provirus expression in response to T cell signaling (19). CDK7 also functions as the CDK-activating kinase for most other CDKs, including those which are directly involved in regulation of cell cycle progression, and, consequently, CDK7 inhibitors typically have broad effects on transcription and cell growth (20, 21).

Like most cellular promoters, RNAPII recruited to the HIV-1 LTR pauses after synthesis of a short nascent leader; the nascent 5´ HIV-1 RNA forms the Tat-responsive (TAR) stem loop structure, which binds the viral transactivator protein Tat (22). Tat recruits postive transription elongation factor-b (P-TEFb) to the viral promoter, consisting of CDK9 and cyclin T1 (23, 24), which phosphorylates serine 2 of RNAPII in addition to the pausing factors 5,6-dichloro-1-ß-D-ribofuranosylbenzimidazole (DRB) sensitivity-inducing factor and negative elongation factor resulting in enhanced transcriptional elongation (25–28). In addition to Tat, the host co-activator tripartite motif containing protein 24 (TRIM24) was shown to promote recruitment of CDK9 to the HIV-1 LTR to stimulate elongation (29, 30). CDK9 inhibitors, including purine derivatives (roscovitine) and flavonoids (flavopiridol), repress HIV-1 expression (31, 32, 33). However, these drugs lack specific activity against CDK9 as they also inhibit CDK2, CDK5, and CDK7, in addition to other cellular enzymes (34, 33), an effect that causes global inactivation of RNAPII and cell cycle arrest (35, 36).

The mediator kinase module, consisting of cyclin C, MED12, MED13, and CDK8 or its paralog CDK19, transiently associates with the core mediator complex at the promoter of signal-responsive genes and selectively regulates transcription by phosphorylating transcription factors (37, 38). CDK8/19 kinase activity can produce both positive and negative effects on transcription, depending on the functional effect of the modification (37, 39, 40). We recently demonstrated that specific chemical inhibitors of CDK8/19 or *CDK8* gene knockout inhibit reactivation of HIV-1 provirus (41), an outcome that is possibly mediated by the effects of CDK8/19 on transcriptional activators that bind the LTR enhancer, including nuclear factor kappa B (NFκB) (42), T cell factor/lymphoid enhancer factor (TCF/LEF) β-catenin (43), and STAT1/3 (44). These observations indicate that CDK8/19 inhibitors could be used for block and lock therapies, but the potential of such compounds for this purpose have not been examined.

Inhibitors of tCDK activity, particularly of CDK9 (45, 46), have been suggested as potential “block and lock” agents (12), but the effectiveness of specific inhibitors of CDK7, CDK9, and CDK8/19 for this purpose has not been established. Recently, more highly specific small molecule inhibitors of CDK7 and CDK9 have been developed (47, 48), and this technological advancement enabled direct comparison of effects caused by specific inhibitors of these kinases on HIV-1 provirus expression, which we describe in this report. We compared the effect of inhibitors of CDK7 (YKL-5-124) (47), CDK9 (LDC000067) (48), and CDK8/19 (Senexin A) (49) on HIV-1 provirus expression. Each of these compounds inhibited HIV-1 provirus expression in model T cell lines and primary CD4^+^ peripheral blood mononuclear cells (PBMCs) isolated from people living with HIV-1. Treatment of cells with CDK9 and CDK8/19 inhibitors was well tolerated over long durations of exposure, whereas CDK7 inhibition caused cell cycle arrest and reduced cell viability. Long-term treatment of HIV-1-infected cells with LDC000067 and/or Senexin A established drug-induced latency. Interestingly, upon removal of drug, we found that latency persisted in cells that had been treated with CDK8/19 inhibitors, while immediate HIV-1 rebound occurred in cells previously treated with the CDK9 inhibitor LDC000067. This indicates that prolonged inhibition of CDK8/19 may produce an epigenetic state on the HIV-1 LTR promoter that is reticent to stochastic reactivation. Overall, these observations present CDK8/19 as a significant potential target for “block and lock” strategies to produce durable HIV-1 proviral latency.

## RESULTS

### tCDK activity is required for HIV-1 induction in response to T cell activation

**Fig 1:**
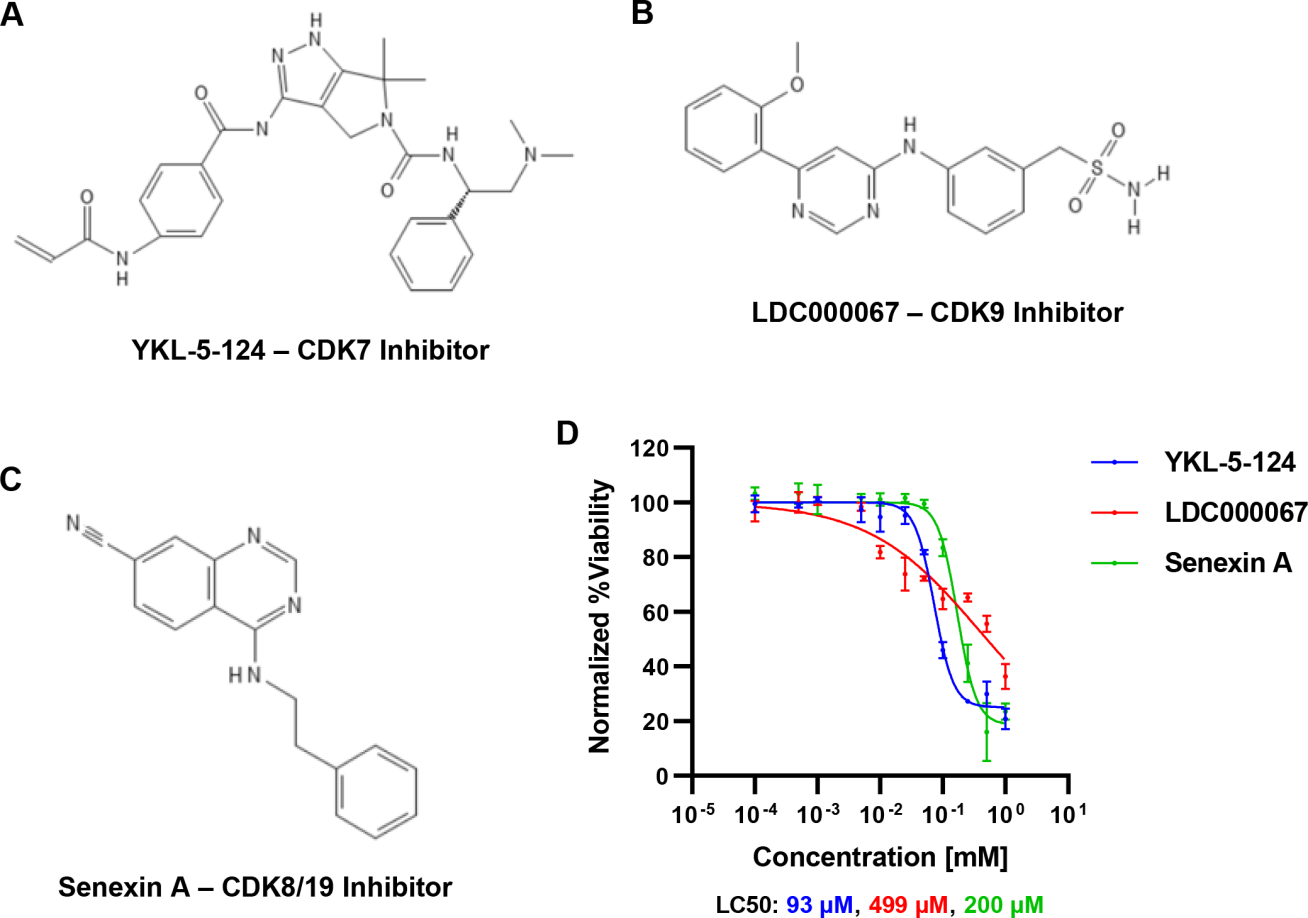
Chemical inhibition of tCDKs is tolerated in T cells.** (A through C)** Molecular structure of YKL-5-124 (CDK7 inhibitor) (**A**), LDC000067 (CDK9 inhibitor) (**B**), and Senexin A (CDK8 and CDK19 inhibitor) (**C**). **(D)** Jurkat human T cells were treated with the concentrations of CDK inhibitor for 24 h, after which cellular viability was determined. Results are normalized to an untreated control (*n* = 2, mean ± SD).

**Fig 2:**
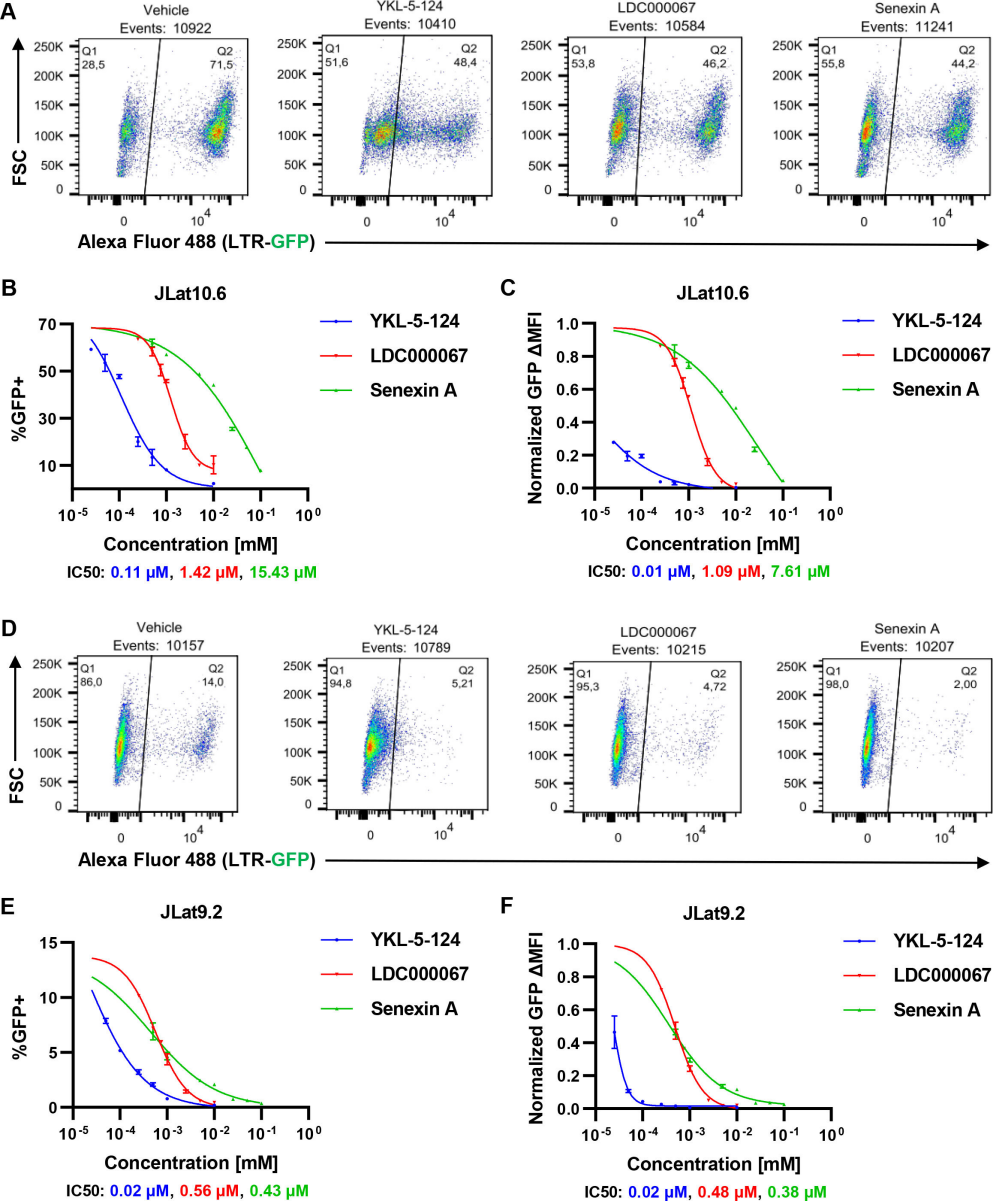
HIV-1 provirus requires tCDK kinase activity for reactivation. (**A)** Representative flow cytometry scatter plots of JLat10.6 cells that were pre-treated for 30 min with dimethyl sulfoxide (DMSO) (vehicle), 100 nM YKL-5-124 (CDK7i), 1 µM LDC000067 (CDK9i), or 10 µM Senexin A (CDK8/19i) and subsequently incubated with 5 nM PMA and 1 µM ionomycin for 20 h.** (B, C)** Following 30-min pre-treatment with the indicated concentration of tCDK inhibitor, JLat10.6 cells were incubated with 5 nM PMA and 1 µM ionomycin. Twenty hours later, flow cytometric analysis was performed to measure HIV-1 expression as reported as the proportion of GFP positive cells (**B**) and the GFP delta (Δ) MFI (**C**) (*n* = 2, mean ±  SD). (**D)** As in (**A**), but JLat9.2 cells were examined. (**E, F)** As in (**B**) and (**C**), but JLat9.2 cells were examined (*n* = 2, mean ±  SD).

We examined the requirement of tCDK activity for HIV-1 expression using the specific small molecule inhibitors YKL-5-124 (CDK7i), LDC000067 (CDK9i), and Senexin A (CDK8/19i) (Fig. 1A through C). These recently developed chemical inhibitors display vastly improved target selectivity over previously described CDK inhibitors (47–49). We assessed the effect of these compounds on cell viability, determining half lethal concentrations (LC50) of 93 µM, 499 µM, and 200 µM for YKL-5-124 (CDK7i), LDC000067 (CDK9i), and Senexin A (CDK8/19i), respectively (Fig. 1D). We then examined the effects of these drugs at non-toxic concentrations on expression of HIV-1 provirus using the well-described JLat10.6 and JLat9.2 models of HIV-1 latency. Derived from the Jurkat human T cell line, JLat cells possess a full-length replication defective (*env*-) provirus where *Nef* is replaced with green fluorescent protein (GFP), such that GFP expression is dependent upon transcriptional activity of the 5´ HIV-1 LTR (50). Treatment with YKL-5-24, LDC000067, or Senexin A caused dose-dependent inhibition of HIV-1 induction in response to phorbol 12-myristate 13-acetate (PMA)/ionomycin in the JLat10.6 cell line (Fig. 2A through C). When calculated as the percentage of cells expressing GFP, we found that YKL-5-124 (CDK7i) was the most potent, with a half inhibitory concentration (IC50) for GFP expression of 0.11 µM, while LDC000067 and Senexin A produced IC50 values of 1.42 µM and 15.43 µM, respectively (Fig. 2B). Comparable results were observed when GFP mean fluorescence intensity (MFI) was used as a readout (Fig. 2C). A similar inhibitory effect was observed with the JLat9.2 cell line (Fig. 2D), although lower concentrations of inhibitor suppressed proviral expression (Fig. 2E and F). Again, the CDK7i YKL-5-124 was the most potent (IC50: 0.02 µM), while LDC000067 and Senexin A had IC50 values of 0.56 µM and 0.43 µM, respectively. Notably, viability of JLat10.6 and JLat9.2 cells were not affected by the tCDK inhibitors at concentrations that impair HIV-1 expression (Fig. S1).

The JLat10.6 and JLat9.2 cell lines, typical of other cell lines bearing HIV-1 reporter provirus (51), produce distinct patterns of GFP reporter expression in response to T cell activation, where ~70% of JLat10.6 and ~14% of JLat9.2 cells fully express GFP upon treatment with PMA and ionomycin, while the remaining cells in the populations remain uninduced (Fig. 2A and D). Treatment of PMA/ionomycin stimulated cells with LDC000067 (CDK9i) or Senexin A (CDK8/19i) diminished the proportion of GFP+ cells (Fig. 2B and E), but caused a similar bimodal distribution of GFP expression, where provirus that escapes latency in response to simulation induce full GFP expression (Fig. 2A and D, LDC000067, Senexin A). In contrast, treatment with the CDK7 inhibitor YKL-5-124 produces a larger gradient of GFP expression in PMA/ionomycin stimulated cells, particularly obvious in the JLat10.6 cell line, where a significant proportion of stimulated cells express lower levels of GFP (Fig. 2A and D, YKL-5-124). These differences in GFP expression patterns in stimulated cells may reflect the distinct effects these inhibitors have on RNAPII activity.

### tCDK inhibitors have synergistic effects on HIV-1 expression

**Fig 3:**
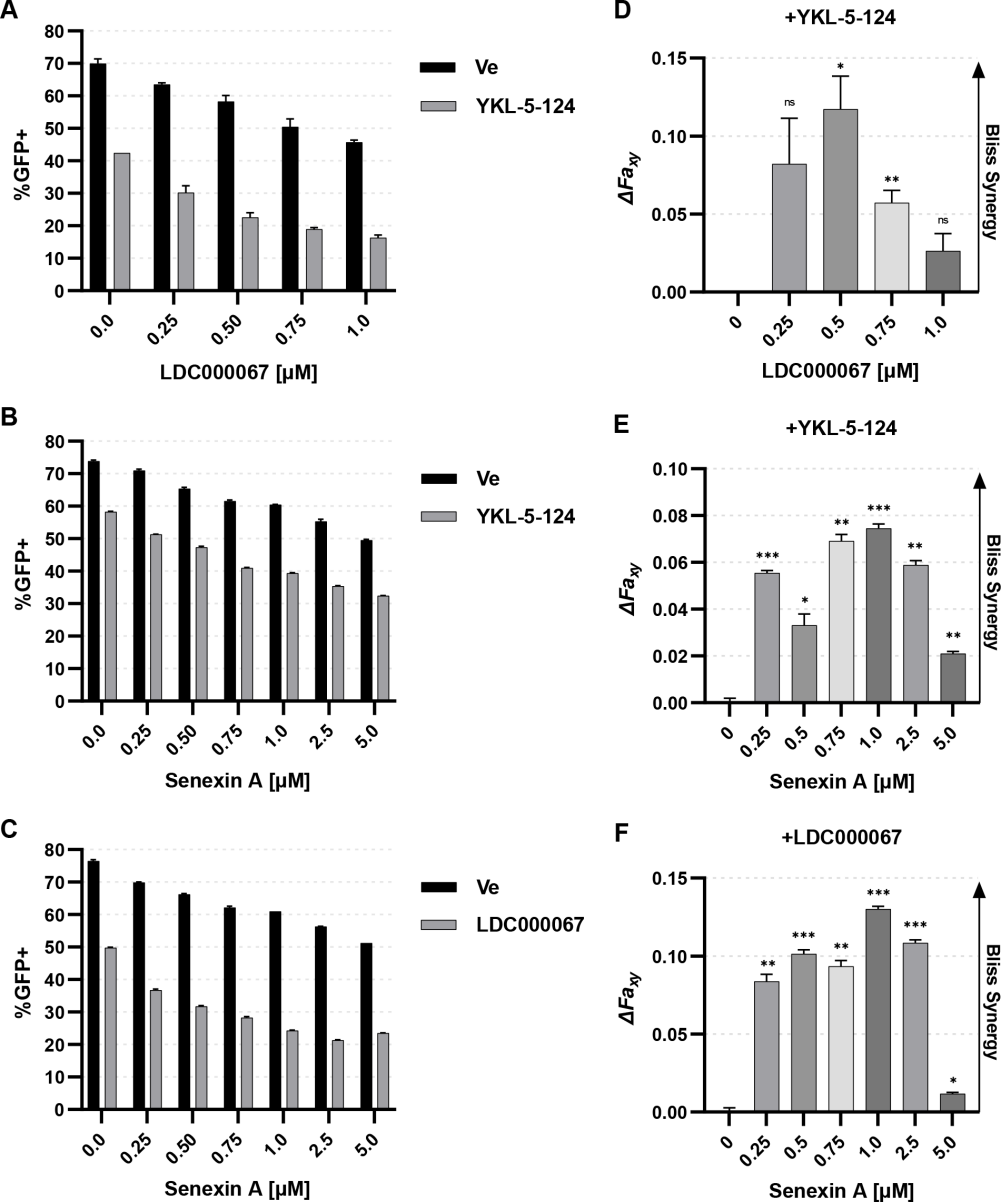
tCDK inhibitors synergistically inhibit HIV-1 transcription. (**A through C)** JLat10.6 cells were pre-treated for 30 min with 100 nM YKL-5-124 (CDK7i) and the indicated concentration of LDC000067 (CDK9i) (**A**) or Senexin A (CDK8/19i) (**B**), or were pre-treated for 30 min with 1 µM LDC000067 and the indicated concentration of Senexin A (**C**). Subsequently, cells were incubated with 5 nM PMA/1 µM ionomycin for 20 h and analyzed by flow cytometry (*n* = 2, mean ±  SD). (**D through F)** Calculation of synergy between the indicated treatments in (A through C) using Bliss independence modeling. Data are presented as the difference between the predicted and the observed fractional HIV-1 expression response to the given drug combination (*n* = 2, mean ±  SD). See Materials and Methods for details.

CDK7, CDK9, and CDK8/19 regulate different aspects of RNAPII function, and consequently, we expected that combined treatment with inhibitors specific for tCDKs would produce synergistic effects for inhibition of HIV-1 provirus expression. To examine this possibility, we treated JLat10.6 cells with YKL-5-124 (CDK7i) and LDC000067 (CDK9i) (Fig. 3A), YKL-5-124 (CDK7i) and Senexin A (CDK8/19i) (Fig. 3B), or LDC000067 (CDK9i) and Senexin A (CDK8/19i) (Fig. 3C), at suboptimal inhibitory concentrations for each, and induced provirus expression by stimulation with PMA and ionomycin. In this analysis, we found that co-treatment with any pair of tCDK inhibitors produced a greater inhibitory effect on HIV-1 expression than individual treatment (Fig. 3A through C), indicating these effects were synergistic. This observation was confirmed by determination of ∆*Fa*_xy_ values which were all greater than 0 as calculated using Bliss independence modeling (Fig. 3D through F) (52–54). Among these combinations, we found that LDC000067 (CDK9i) and Senexin A (CDK8/19i) produced the greatest synergistic inhibition of HIV-1 reactivation (Fig. 3D through F). The synergistic inhibitory effect for all combinations was most pronounced at lower drug concentrations, and decreased at the highest concentrations, where GFP expression was minimal (Fig. 3D through F). These observations indicate selectivity of the tCDK inhibitors for CDK7, CDK9, or CDK8/19, and suggest that these kinases function through independent mechanisms for regulation of RNAPII. Importantly, cell viability was not affected at inhibitor concentrations where we observed synergistic inhibitory effects on HIV-1 provirus expression (Fig. S2).

### tCDK inhibitors antagonize proviral reactivation in response to mechanistically diverse latency reversal agents

**Fig 4:**
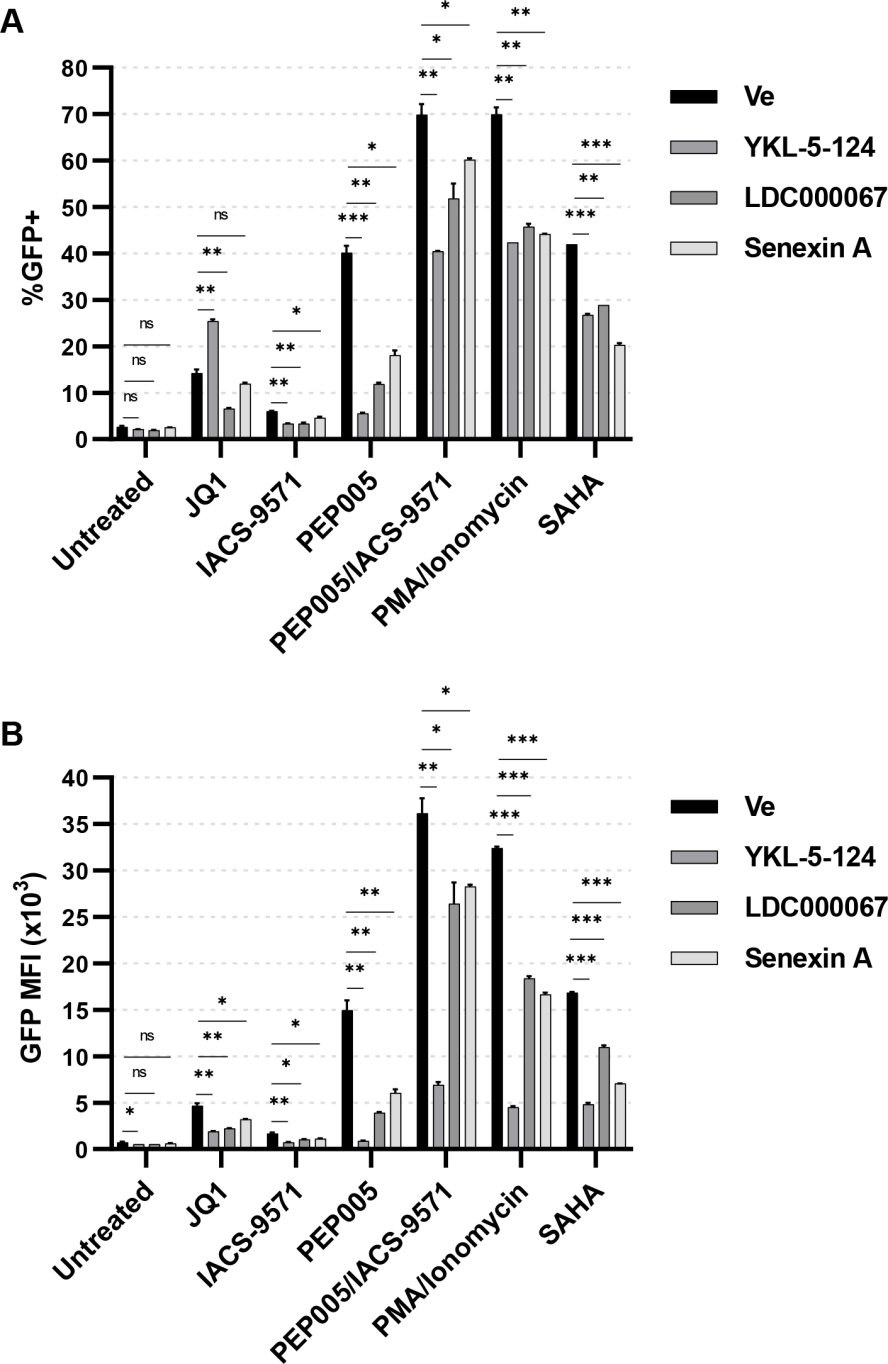
tCDK kinase inhibitors antagonize the effect of latency reversing agents.** (A, B)** JLat10.6 cells were pre-treated for 30 min with 100 nM YKL-5-124 (CDK7i), 1 µM LDC000067 (CDK9i), 10 µM Senexin A (CDK8/19i), or a vehicle control (Ve, DMSO). Subsequently, cells were incubated with 10 µM JQ1, 20 µM IACS-9571, 5 nM PEP005, 5 nM PEP005/20 µM IACS-9571, 5 nM PMA/1 µM ionomycin, 5 µM SAHA, or were left untreated. Following 20 h, GFP expression was measured by flow cytometry and reported as the percent of GFP positive cells (**A**) and the MFI of GFP (**B**) (*n* = 2, mean ±  SD).

Having observed that tCDK inhibition suppresses reactivation of latent provirus in response to T cell activation, we next examined their effects on a variety of mechanistically diverse LRAs. As mentioned above, co-treatment with the phorbol ester PMA and the ionophore ionomycin triggers T cell activation by stimulating Ras-mitogen activated protein kinase (MAPK), protein kinase C (PKC)-NFκB, and calcineurin-nuclear factor of activated T cells (NFAT) (55). Ingenol 3-angelate (PEP005) activates PKC-NFκB, partially mimicking T cell activation (56). JQ1 and IACS-9571 are bromodomain inhibitors that reverse latency by interfering with function of their respective targets, bromodomain containing protein 4 (BRD4) (57) and TRIM24 (29). Finally, suberoylanilide hydroxamic acid (SAHA) is a well-studied histone deacetylase inhibitor (HDACi) known to reactivate HIV-1 expression *in vitro* and *in vivo* (58, 59). Of these LRAs, the bromodomain inhibitors JQ1 and IACS-9571 induced only modest proviral reactivation in the JLat10.6 cell line (Fig. 4A and B, black bar). In contrast, PMA/ionomycin, the NFκB agonist PEP005, alone or in combination with IACS-9571, and the HDACi SAHA produced markedly higher levels of HIV-1 provirus expression (Fig. 4A and B).

To examine the effect of tCDK inhibitors on reactivation by these LRAs, we co-treated cells with concentrations near the IC50 for inhibition of PMA/ionomycin-induced expression in JLat10.6 cells, which was 100 nM YKL-5-124 (CDK7i), 1 µM LDC000067 (CDK9i), or 10 µM Senexin A (CDK8/19i). CDK7 inhibition by YKL-5-124 limited proviral reactivation, as measured by the proportion of GFP+ cells, in response to every LRA other than JQ1 where, oddly, we observed an increased proportion of GFP expressing cells (Fig. 4A). Interestingly, although inhibition of CDK7 produced a greater proportion of GFP+ cells when treated with JQ1 (Fig. 4A), the GFP MFI value was decreased compared to the vehicle control (Fig. 4B), indicating that more cells expressed GFP but at attenuated levels in the presence of YKL-5-124. Treatment with LDC000067 (CDK9i) or Senexin A (CDK8/19i) inhibited reactivation of HIV-1 in response to all LRAs (Fig. 4A and B). Consistent with previous observations (29), we observed a synergistic effect for HIV-1 reactivation by co-treatment with the NFκB agonist PEP005 and the TRIM24 bromodomain inhibitor IACS-9571 (Fig. S3). Moreover, tCDK inhibitors were the least effective at preventing reactivation of provirus in cells treated with the combination of PEP005 and IACS-9571 (Fig. 4A and B). Finally, although the tCDK inhibitors suppressed the appearance of GFP positive cells in response to PMA/ionomycin treatment by approximately equivalent amounts (Fig. 4A, PMA/ionomycin), certain inhibitors were more effective at inhibiting reactivation by particular LRAs. For instance, Senexin A (CDK8/19i) was most effective at suppressing SAHA-mediated activation (Fig. 4A, SAHA) while LDC000067 (CDK9i) was most potent at inhibiting expression in response to JQ1 (Fig. 4A, JQ1). Taken together, these results indicate that tCDK inhibition suppresses proviral activation in response to mechanistically diverse latency reversing agents.

### Requirement of Tat for the effect of tCDKs on HIV-1 transcription

**Fig 5:**
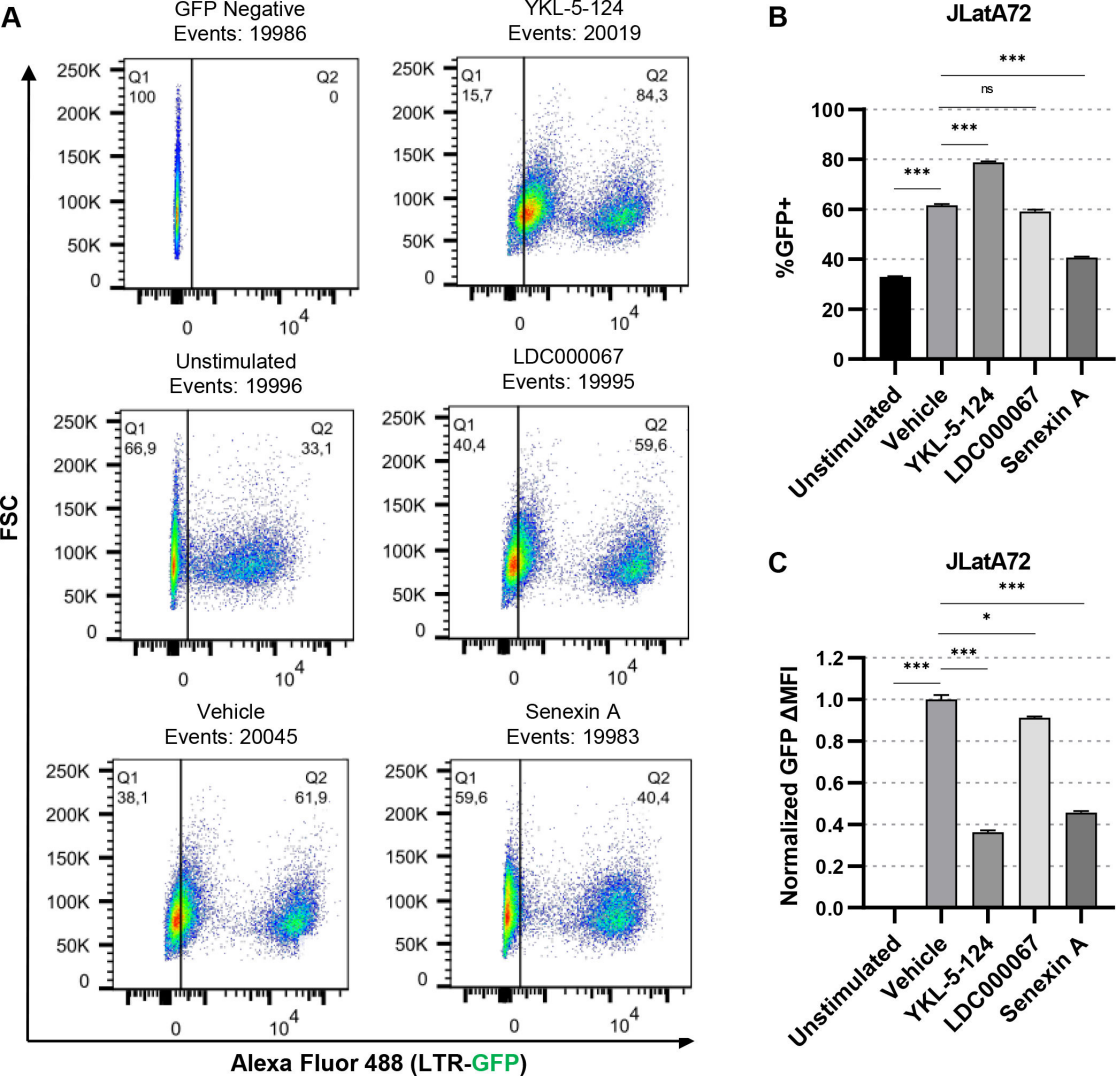
Requirement of Tat for regulation of HIV-1 expression by tCDKs. (**A)** Representative flow cytometry scatter plots of JLatA72 Jurkat cells (-Tat), pre-treated for 30 min with a vehicle control (DMSO), 100 nM YKL-5-124 (CDK7i), 1 µM LDC000067 (CDK9i), or 50 µM Senexin A (CDK8/19i) and subsequently incubated with 5 nM PMA and 1 µM ionomycin for 20 h.** (B, C)** JLatA72 cells were left untreated or pre-treated for 30 min with a vehicle control (DMSO), 100 nM YKL-5-124, 1 µM LDC00067, or 50 µM Senexin A prior to stimulation with 5 nM PMA/1 µM ionomycin. Following 20 h, flow cytometry was performed with viral expression reported as the percent of GFP positive cells (**B**) and the delta GFP MFI (**C**) (*n* = 2, mean ±  SD).

We then examined the dependence of CDK7, CDK9, and CDK8/19 on the viral transactivator Tat for activation of HIV-1 transcription. The Jurkat-derived JLatA72 cell line possesses an integrated HIV-1 LTR that drives expression of GFP but, unlike the JLat10.6 and JLat9.2 cell lines, does not express the viral Tat protein (60). Untreated JLatA72 cells express GFP in ~30% of the population, and this proportion increases to ~60% upon treatment with PMA/ionomycin (Fig. 5A and B, unstimulated, vehicle). To examine the effect of tCDK inhibitors on HIV-1 expression in the absence of Tat, we treated JLatA72 cells with the kinase inhibitors and subsequently induced T cell activation with PMA/ionomycin. Surprisingly, we found that inhibition of CDK7 with YKL-5-124 caused an increase in the proportion of GFP positive cells in stimulated cells (Fig. 5A and B) while also lowering overall proviral expression in these cells (Fig. 5C), an effect that is similar to that described above for co-treatment with JQ1 (Fig. 4). Notably, treatment of unmodified Jurkat cells with YKL-5-124, alone or in combination with PMA and ionomycin, does not cause autofluorescence (Fig. S4), indicating that increases in fluorescence in treated reporter lines is indicative of HIV-1 GFP expression in these cells. The CDK9 inhibitor LDC000067 had no effect on the proportion of GFP positive cells (Fig. 5A and B) and only slightly decreased overall GFP expression (Fig. 5C), consistent with the role of Tat for recruitment of CDK9 to the HIV-1 LTR. Inhibition of CDK8/19 with Senexin A decreased both the proportion of cells expressing GFP in response to PMA/ionomycin (Fig. 5A and B), and suppressed overall levels of GFP expression in induced cells (Fig. 5C), indicating that the effect of CDK8/19 on reactivation of HIV-1 expression is not solely dependent on Tat.

We also examined the effect of tCDK inhibitors in the CEM-derived GXR-5 cell line, which like JLatA72, possess a chromosomally integrated HIV-1 LTR GFP reporter that does not express Tat (61). However, unlike JLatA72 cells, GXR-5 cells produce a basal level of provirus expression such that most untreated cells express some amount of GFP (Fig. S5A and B). However, stimulation with PMA/ionomycin causes an increase in GFP expression as measured by MFI (Fig. S5C). Similar to results with the JLatA72 cell line, GXR-5 cells produced significantly attenuated induction of GFP upon treatment with YKL-5-124 (CDK7i) or Senexin A (CDK/19i) (Fig. S5C). In contrast, LDC000067 (CDK9i) caused a small but significant inhibition of GFP expression (Fig. S5C), which supports the requirement of CDK9 for Tat function.

### Effect of tCDK inhibition on cell growth

**Fig 6:**
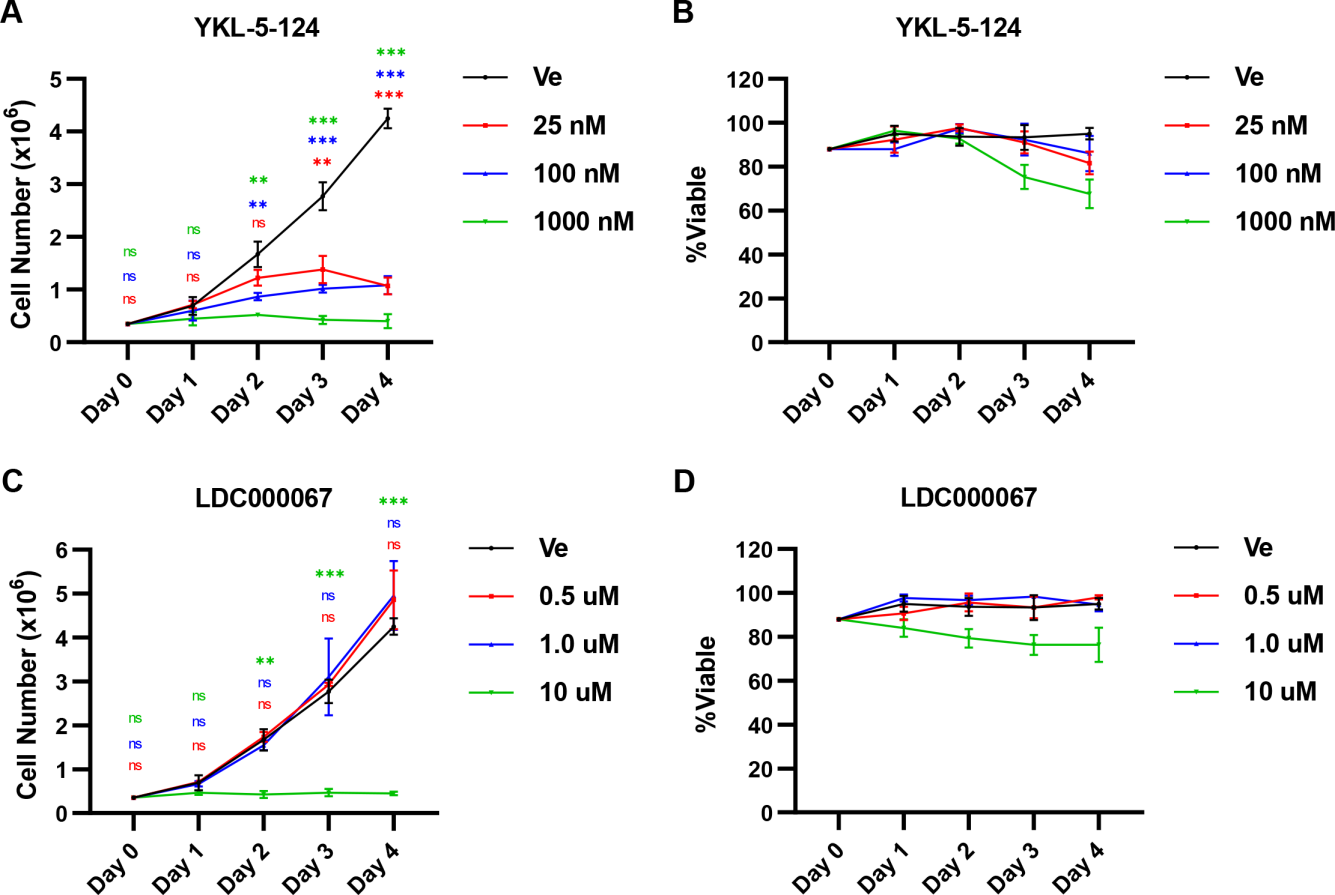
Effect of sustained tCDK inhibition on cell growth and viability. (**A, B)** Jurkat T cells were incubated with the indicated concentration of YKL-5-124 or a vehicle control (Ve, DMSO) for the indicated time (days), total cell count (**A**), and viability (**B**) were determined. Media was replaced daily with inhibitor (*n* = 3, mean ±  SD). (**C, D)** As in (**A**) and (**B**), but cells were treated with LDC000067 (*n* = 3, mean ±  SD).

The results presented above indicate that tCDK activity is necessary for reactivation of HIV-1 provirus. Given the stochastic expression phenotype observed for chromosomally integrated provirus (51, 62, 63), therapies intended to prevent HIV-1 reactivation will likely necessitate prolonged treatment to produce latency that could persist subsequent to drug withdrawal. To further examine the cellular impact of tCDK inhibition, we examined growth and viability of Jurkat T cells exposed to a range of tCDK inhibitor concentrations for 4 days. This revealed that concentrations of YKL-5-124 (CDK7i) that inhibited HIV-1 provirus expression completely prevented cell growth and caused cell cycle arrest (Fig. 6A), a result that is consistent with the defined function of CDK7 as the CDK-activating kinase (CAK) which phosphorylates and activates CDKs that have essential roles for cell cycle progression (64). Moreover, treatment with YKL-5-124 causes toxicity that becomes apparent after 3 or 4 days of treatment, depending on the concentration (Fig. 6B). In contrast, the CDK9 inhibitor LDC000067 caused no discernable effects on cell growth (Fig. 6C) and cell viability (Fig. 6D) at concentrations that limit HIV-1 provirus expression, although cell division was prevented, and toxicity became apparent at higher concentrations. Similar to CDK9 inhibition, we have previously found that the CDK8/19 inhibitor Senexin A does not produce toxic effects or inhibit cell growth at concentrations that suppress HIV-1 transcription (41). These observations indicate that long-term inhibition of CDK9 and/or CDK8/19, but not CDK7, may be tolerated for suppression of HIV-1 provirus reactivation.

### Role of tCDKs for the establishment of immediate latency

**Fig 7:**
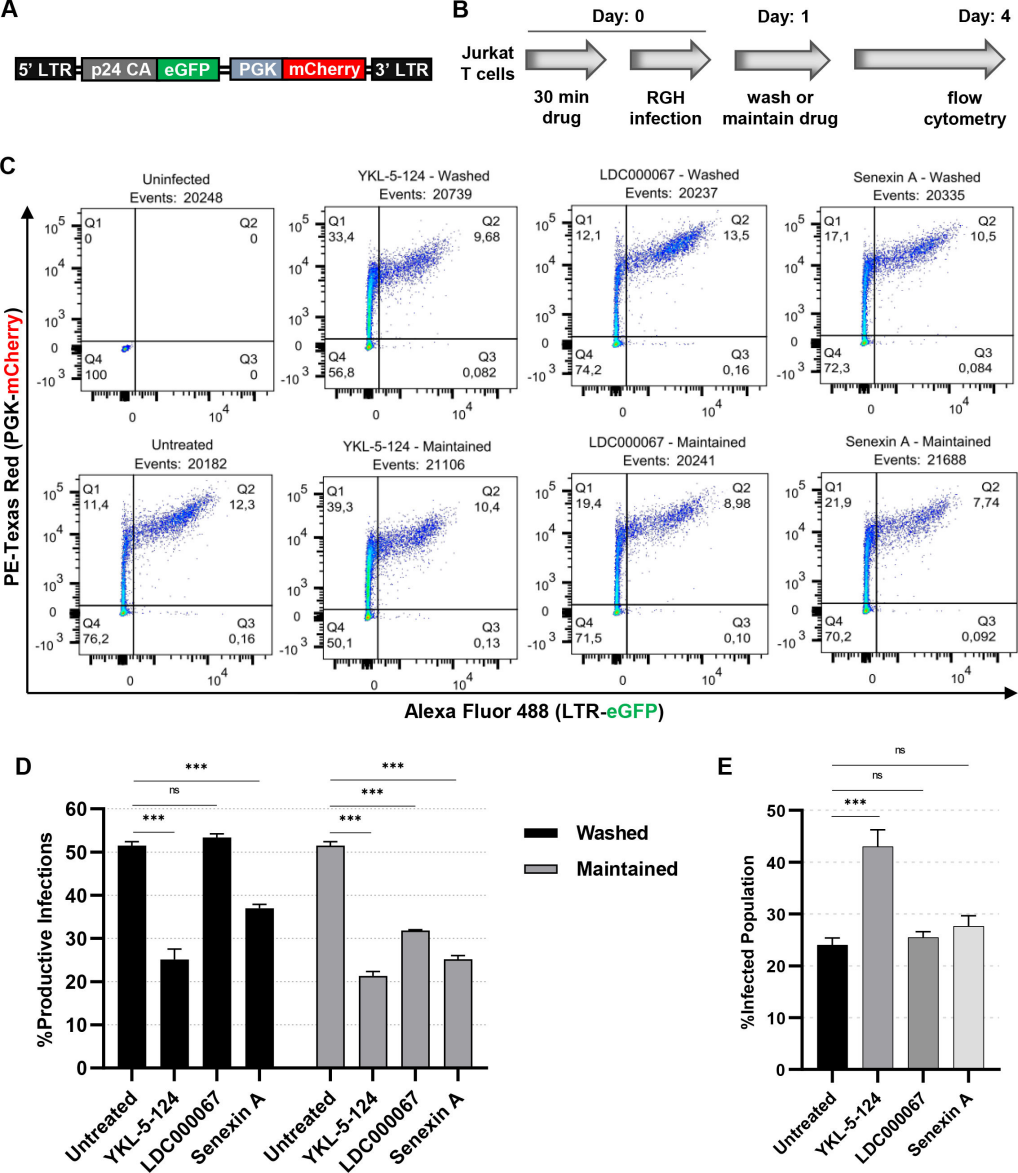
Inhibition of tCDK activity encourages establishment of latency upon infection. (**A)** Simplified depiction of the replication incompetent RGH dual reporter virus (66), which expresses GFP from the 5´ LTR and mCherry from an internal constitutive PGK promoter. (**B)** Jurkat CD4^+^ human T cells were left untreated or were pre-treated for 30 min with 100 nM YKL-5-124, 1 µM LDC000067, or 10 µM Senexin A prior to infection of cells with RGH. One day post-infection, media was replaced, and cells were either washed of the drug or maintained with the same concentration of inhibitor. Four days post-infection, proviral expression was assessed by flow cytometry. (**C)** Representative flow cytometry scatter plots of RGH-infected cells, treated as indicated in (**B**) and examined 4 days post-infection. Q4 contains the uninfected (fluorescent negative) cells, Q1 is the population of latently infected cells (mCherry+/GFP-), Q2 displays the productive infections (mCherry+/GFP+), while Q3 contains noise generated from viral rearrangements (mCherry-/GFP+). (**D)** Jurkat T cells were treated as in (**B**), with the percentage of productive infections determined by flow cytometry 4 days post-infection. The percentage of productive infections was determined from the ratio of GFP+/mCherry+ cells to all mCherry+ cells (*n* = 3, mean ±  SD). (**E)** Summary of the infection rate for cells treated as indicated, representing the proportion of cells expressing mCherry (*n* = 3, mean ±  SD).

Upon infection of T cells with HIV-1, nearly 50% of newly integrated provirus become transcriptionally repressed within 48 h post-infection, despite the site of chromosomal integration, a phenomenon known as immediate latency (65–67). Because the tCDKs are required for reactivation of HIV-1 in response to various LRAs and T cell signaling agonists, we also examined the effect of these inhibitors on outcome of initial infection by HIV-1. For this analysis, we used the Red-Green-HIV-1 (RGH) dual-reporter virus (Fig. 7A) (65, 66), which expresses GFP from the 5´ LTR and mCherry from an internal promoter, and produces all of the HIV-1 gene products apart from *env* and *nef* (66). Upon infection with the RGH reporter virus, cells that express mCherry from the phosphoglycerate kinase (PGK) promoter, but that do not express GFP from the 5´ HIV-1 LTR, represent latently infected cells, while those that express both mCherry and GFP are productively infected (65, 66). To assess the role of tCDK activity on establishment of immediate latency, we pre-treated Jurkat CD4^+^ T cells with the kinase inhibitors and subsequently infected with RGH at a low multiplicity of infection (MOI). One day post-infection, the cells were either washed to remove residual drug or were maintained in media with the kinase inhibitors, and proviral expression was assessed 4 days post-infection by flow cytometry (Fig. 7B and C). Inhibition of either CDK7 with YKL-5-124 or CDK8/19 with Senexin A at the time of infection reduced the proportion of productively infected cells 4 days later, whereas inhibition of CDK9 with LDC000067 had no effect (Fig. 7D, washed), indicating that CDK9 and Tat are not involved in the decision to establish latency at the time of infection. Consistent with the results shown above demonstrating that tCDK inhibition prevents reactivation of latent provirus (Fig. 2 and 4), maintained treatment with the kinase inhibitors post-infection decreased the proportion of productively infected cells, indicating that these drugs encourage latency (Fig. 7D, maintained).

Surprisingly, we found that inhibition of CDK7 with YKL-5-124 caused approximately double the rate of infection, suggesting that loss of CDK7 activity may render T cells susceptible to infection (Fig. 7E). Notably, although CDK7 inhibition caused an increase in the proportion of HIV-1-infected cells (Fig. 7E), the infected population is enriched for latent provirus, as just over 20% were productively infected (Fig. 7C and D, compare untreated to YKL-5-124). Importantly, kinase inhibition did not affect cellular viability under these conditions (Fig. S6). These results reveal that the inhibition of CDK7 or CDK8/19 at the point of infection inhibits formation of productive infection after removal of the drug, while inhibition of CDK9 does not have this lasting effect. Although the CDK7 inhibitor YKL-5-124 has a latency promoting effect in newly infected cells, it has associated shortcomings of inducing cell cycle arrest (Fig. 6A) and rendering cells susceptible to infection (Fig. 7E).

### Inhibition of CDK8/19 but not CDK9 induces durable latency.

**Fig 8:**
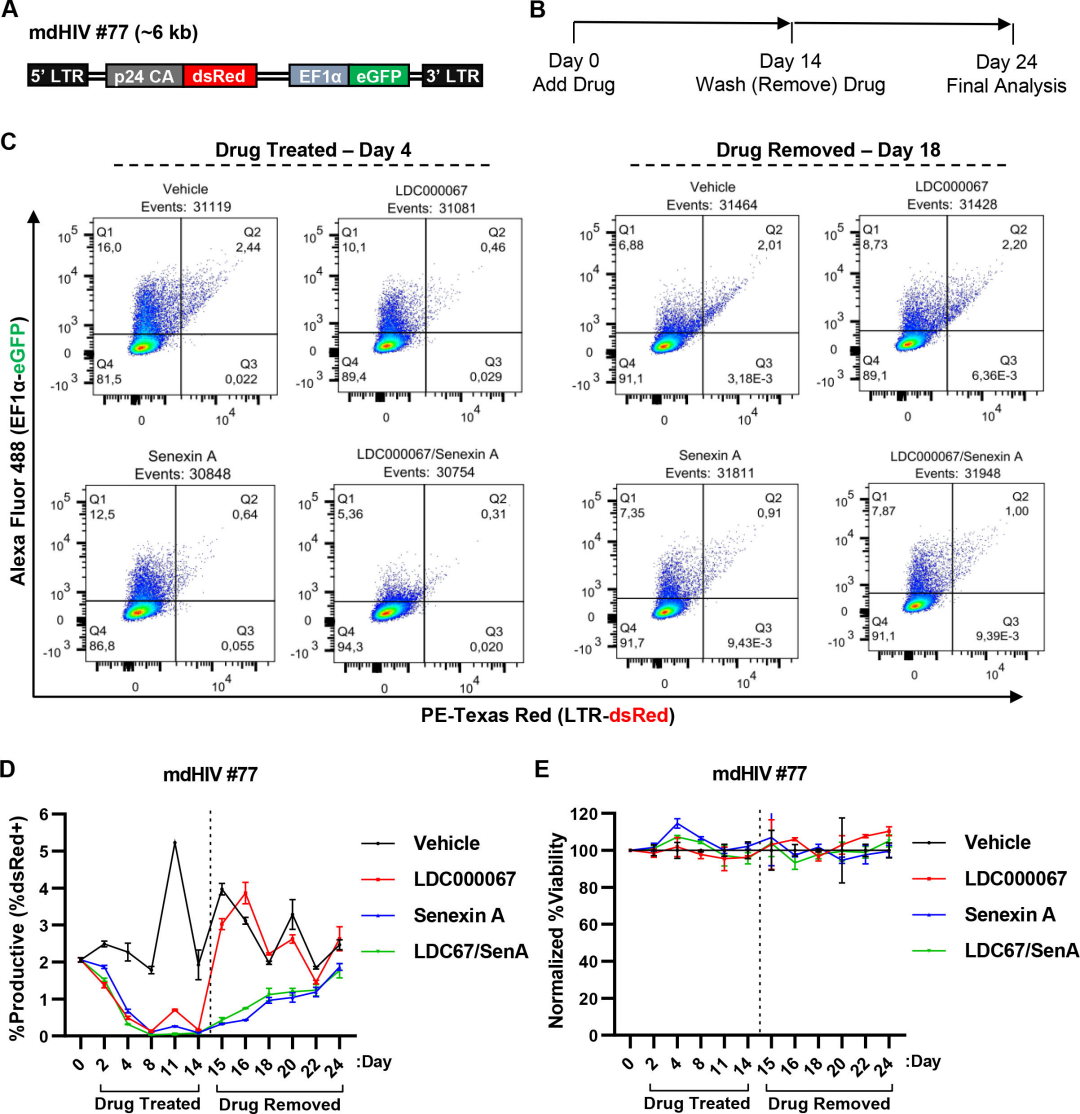
Effect of CDK9 and CDK8/19 inhibitors on basal expression of an HIV-1 mini-virus. (**A)** Simplified schematic depiction of the mini-dual HIV-1 reporter virus in the mdHIV #77 Jurkat Tat clonal T cell line (51), which expresses dsRed from the 5´ LTR and eGFP from an internal EF1α promoter. (**B)** mdHIV #77 cells were incubated with a vehicle control (DMSO), 1 µM LDC000067, 10 µM Senexin A, or 1 µM LDC000067 and 10 µM Senexin A for 14 days, when the drug(s) were removed, and HIV-1 expression was assessed by flow cytometry over the indicated time (days). (**C)** Representative flow cytometry scatter plots following 4 days of drug treatment followed by 4 days after drug withdrawal. Each dot indicates a single HIV-1-infected cell, where the population in Q2 represents cells with transcriptionally active provirus. (**D)** mdHIV #77 cells were treated as in (**B**) with viral expression assessed on the indicated day by flow cytometry (*n* = 2, mean ±  SD). (**E)** mdHIV #77 cells treated as indicated in (**B**) were examined for viability, with values normalized to the vehicle control (*n* = 2, mean ±  SD).

An optimal “block and lock” therapy would involve treatment(s) that induce durable latency, even after the LPA is removed. To examine the capability of the tCDK inhibitors to induce durable latency, we assessed the effect of these compounds on the mdHIV #77 cell line (51), which is derived from the Jurkat Tat cell line and bears a replication incompetent mini-dual reporter HIV-1 provirus that is integrated into an intron of the *PACS1* gene in the (+) orientation (Fig. 8A). HIV-1 reporter cell lines typically produce stochastic bursts of HIV-1 expression (51, 62), and consistently ~3% of untreated mdHIV #77 cells co-express dsRed from the HIV-1 5´ LTR and eGFP from an internal elongation factor 1 alpha (EF1α) promoter (Fig. 8C, vehicle) (51). To examine the effect of tCDK inhibitors on stochastic expression of latent HIV-1 provirus, we treated mdHIV #77 cells for 14 days with the tCDKi, after which the cells were washed, and the drug treatments were discontinued. Expression of dsRed from the 5´ LTR was monitored for 10 days following drug removal by flow cytometry (Fig. 8B and C). The effect of the CDK7 inhibitor YKL-5-124 (CDK7i) in this experiment was not assessed because of associated toxicity (Fig. 6A and B).

In these experiments, we observed that stochastic HIV-1 expression was suppressed in this cell line by treatment with LDC000067 (CDK9i) and/or Senexin A (CDK8/19i) following 4 days of treatment (Fig. 8C and D). Following removal of the CDK inhibitors, we found that cells that had been treated with LDC000067 (CDK9i) produced an immediate rebound of provirus expression, as the population of dsRed+/eGFP+ cells nearly matched that of untreated cells within 24 h of drug withdrawal (Fig. 8D, compare day 14 to 15). In contrast, mdHIV #77 cells that had been treated with Senexin A (CDK8/19i) alone, or in combination with LDC000067 (CDK9i), maintained decreased levels of dsRed expression for at least 7 days following drug removal (Fig. 8D, drug removed). Importantly, none of the inhibitors affected cell viability over the course of treatment (Fig. 8E). We observed similar results with the Jurkat Tat mdHIV #110 clonal line (Fig. S7). This line has the mini-dual HIV-1 reporter integrated in the (-) orientation in the *BTBD10* gene, and produces stochastic dsRed expression in ~25% of untreated cells (51) (Fig. S7C, vehicle). Treatment of mdHIV #110 cells with the CDK9 and CDK8/19 inhibitors suppressed HIV-1 LTR dsRed expression (Fig. S7D, drug treated). But removal of drug from cells treated with LDC000067 (CDK9i) resulted in immediate rebound of dsRed expression, whereas dsRed expression gradually increased over the week following removal of drug(s) from cells treated with Senexin A (CDK8/19i), alone or in combination with LDC000067 (Fig. S7D, drug removed). Again, we did not observe effects on viability of mdHIV #110 cells over the course of tCDKi treatment (Fig. S7E).

Given the finding that treatment with the CDK8i Senexin A caused suppression of proviral expression, an effect that persists for several days following removal of the drug (Fig. 8; Fig. S7), we sought to determine if this effect is distinct to Senexin A or is a property of CDK8/19 inhibitors in general. To examine this, we treated the mdHIV #77 cell line with Senexin A or the structurally unrelated CDK8/19 inhibitor BRD6989 (68). Here, we maintained cells in culture with either inhibitor for 7 days and then removed the drug from media by washing and examined expression of the dsRed reporter over the following 10 days (Fig. S8A). We found that both CDK8/19 inhibitors reduced the proportion of cells that expressed HIV-1, with Senexin A displaying a slightly stronger inhibitory effect than BRD6989 (Fig. S8B and C). Upon removal of the drugs, the suppressive effect on HIV-1 expression endured for several days in cells treated with either Senexin A or BRD6989 (Fig. S8C). Neither of the CDK8 inhibitors affected cell viability during treatment (Fig. S8D). These results display that two structurally unrelated CDK8 inhibitors Senexin A and BRD6989 produce similar persistent repressive effects on provirus expression, indicating that inhibition of the mediator kinases produces repressive environment on the HIV-1 LTR that persists following removal of the drugs. Furthermore, treatment of cells with Senexin A for either 7 or 14 days, caused similar timing of HIV-1 provirus expression rebound following removal of the drug (Fig. 8; Fig. S8), which indicates that more prolonged treatment with the inhibitor does not produce a corresponding prolonged delay of reactivation. One possibility is that, because treatment with Senexin A does not affect cell growth (41), reactivation of stochastic HIV-1 expression upon removal of the drug may require cell division.

**Fig 9:**
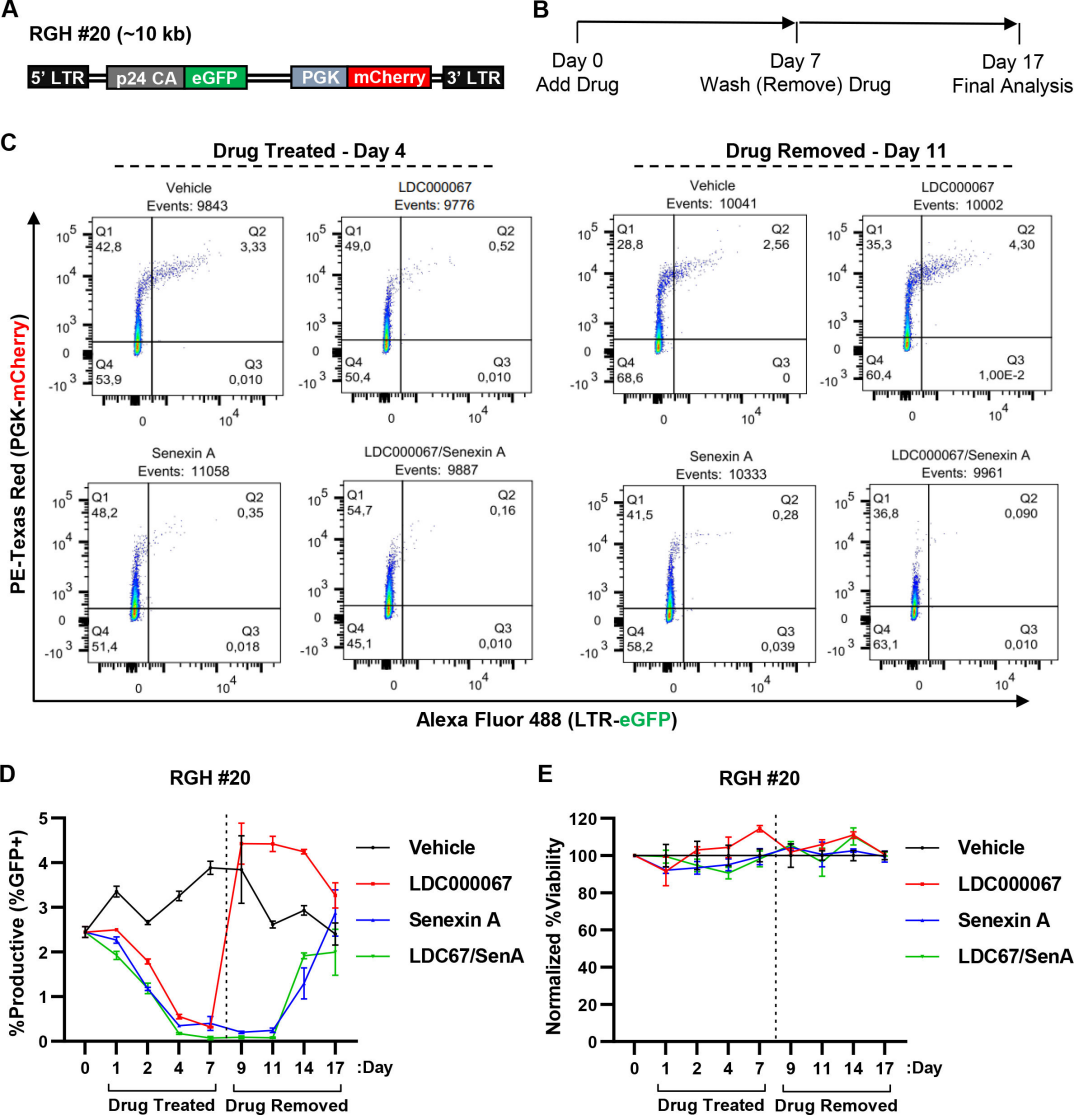
Effect of CDK9 and CDK8/19 inhibitors on RGH proviral latency. (**A)** Simplified schematic depiction of the full-length, replication incompetent HIV-1 RGH reporter virus chromosomally integrated in the RGH #20 Jurkat clonal T cell line, where eGFP is expressed from the 5´ LTR, and mCherry from an internal PGK promoter that replaces *Nef*. (**B)** RGH #20 cells were incubated with a vehicle control (DMSO), 1 µM LDC000067, 10 µM Senexin A, or 1 µM LDC000067 and 10 µM Senexin A for 7 days, when drug(s) were removed, and viral expression was measured by flow cytometry for 10 more days. (**C)** Representative flow cytometry scatter plots following 4 days of drug treatment and 4 days following drug withdrawal. All “dots” are indicative of a single HIV-1-infected cell with Q2 (eGFP+/mCherry+) displaying cells with transcriptionally active provirus. (**D)** RGH #20 cells were treated as in (**B**) with viral expression assessed on the indicated day by flow cytometry (*n* = 2, mean ±  SD). (**E)** RGH #20 cells treated as indicated in (**B**) were examined for viability, with values normalized to the vehicle control (*n* = 2, mean ±  SD).

The Jurkat Tat cell lines bearing the mdHIV reporters constitutively express Tat protein, but the integrated proviruses do not express any viral gene products (51). To examine the effect of the tCDK inhibitors on HIV-1 provirus in more detail, we produced a clonal Jurkat T cell line bearing an integrated RGH reporter virus, which encodes all of the viral genes apart from *env* and *nef,* and where Tat expression is dependent upon normal expression from a subgenomic HIV-1 transcript (Fig. 9A, RGH #20). These cells were treated for 7 days with LDC000067 (CDK9i) and/or Senexin A (CDK8/19i), at which point the cells were washed and GFP expression was monitored for 10 days following drug removal (Fig. 9B). Treatment of RGH #20 cells with the tCDK inhibitors suppressed provirus expression within 4 days (Fig. 9C and D, drug treated). Consistent with the results described above with the mdHIV cell lines, we found that removal of drug from RGH #20 cells treated with the CDK9 inhibitor LDC000067 resulted in immediate rebound of LTR-driven GFP expression, indicating reactivation of basal HIV-1 transcription. In contrast, in cells treated with the CDK8/19 inhibitor Senexin A, alone or in combination with LDC000067, we observed suppression of HIV-1 GFP expression for 4 days following removal of the drug(s), and full rebound of expression was not observed until 10 days later (Fig. 9D, drug removed). As with the experiments described above, we did not observe effects on cell viability in treatment of the RGH #20 cell line (Fig. 9E). These results suggest that CDK8/19 inhibitors, but not inhibitors of CDK9, induce a repressive configuration of the HIV-1 promoter that persists following removal of treatment.

### tCDK inhibitors suppress proviral expression in cells from people living with HIV-1 *ex vivo*

**Fig 10:**
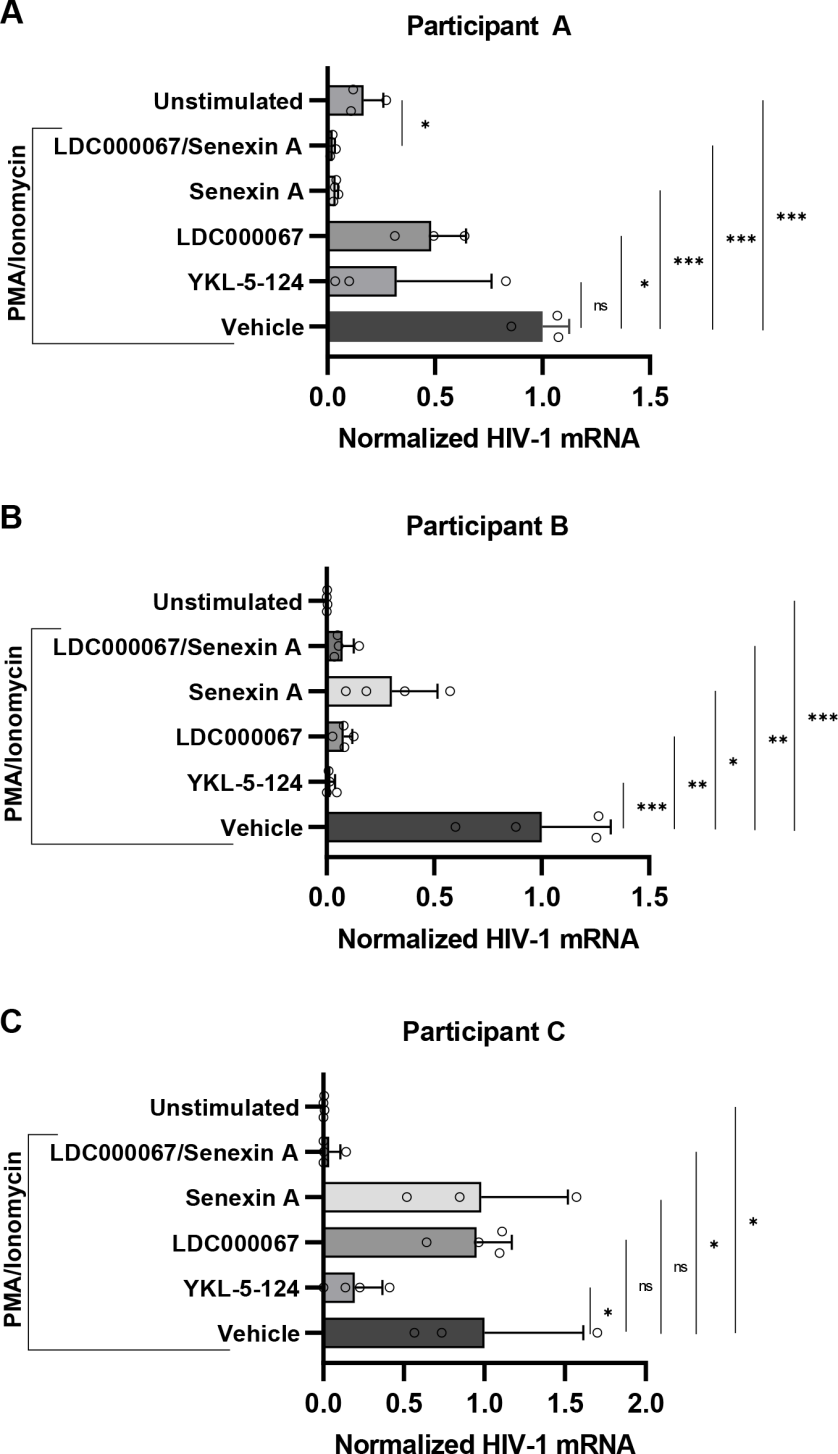
tCDK activity is required for reactivation of HIV-1 in CD4^+^ PBMCs *ex vivo*. (A–C) PBMCs isolated from HIV-1-infected individuals on ART were left untreated or pre-treated for 30 min with vehicle (DMSO), 100 nM YKL-5-124, 1 µM LDC000067, 10 µM Senexin A, or 1 µM LDC000067/10 µM Senexin A. Following pre-treatment, cells were incubated with 5 nM PMA/1 µM ionomycin for 24 h, after which intracellular RNA was extracted and analyzed using RT-PCR with oligos specific for multiply spliced Tat-Rev HIV-1 mRNA transcripts. HIV-1 mRNA expression is normalized to *GAPDH* (*n* = 3–4, mean ±  SD).

To directly compare effectiveness of tCDK inhibitors on HIV-1 provirus expression in normal human CD4^+^ cells, we examined the effect of these compounds on reactivation of provirus in primary PBMCs isolated from individuals living with HIV-1 and receiving suppressive antiretroviral therapy (Table 1). In cells from these individuals, we observed increased HIV-1 mRNA in response to PMA/ionomycin treatment (Fig. 10, compare unstimulated to vehicle). Consistent with the above results, treatment with YKL-5-124 (CDK7i) decreased provirus expression in all three participants, while treatment with LDC000067 (CDK9i) or Senexin A (CDK8/19i) decreased provirus expression in two of three participants (Fig. 10). Interestingly, the combination of LDC000067 and Senexin A produced the most robust silencing of viral expression in all three participants (Fig. 10, LDC000067/Senexin A), with Participant A displaying even lower expression of HIV-1 mRNA than when left unstimulated (Fig. 10A). Furthermore, for Participant C, we observed synergistic inhibition of expression with LDC000067 and Senexin A co-treatment (Fig. 10C). These results indicate that tCDK inhibition can suppress proviral reactivation in cells from individuals living with HIV *ex vivo*.

**TABLE 1:** Participant reservoir characteristics

Participant	Sex	Intact HIV DNA copies per million CD4^+^ T cells^*a*^	Total HIV DNA copies per million CD4^+^ T cells^*a*^	% Intact HIV copies^*b*^
A	Male	340	4,941	6.9
B	Male	397	3,775	10.5
C	Male	197	4,858	4.1

^*a*^ Measured using the intact proviral DNA assay (69).

^*b*^ Percentage of all HIV DNA copies that are genetically intact (calculated as intact copies / total copies * 100%).

## DISCUSSION

Development of therapeutic strategies to eliminate the life-long requirement of ART for people living with HIV-1 currently includes those aimed at modulating expression of the latent provirus (3). The proposed “shock and kill” strategy for elimination of latently infected cells has been one focus of intense investigation, and although a variety of latency reversing agents have been identified (6, 70), none have successfully reduced the latent population in infected individuals in trials (71), largely because of an inefficient anti-HIV-1 cellular immune response (4, 70, 72, 73). The antithetical “block and lock” strategy may be a more feasible approach toward treatment of the latently infected population for functional elimination of HIV-1, and this contention is supported by results shown here. Block and lock would involve use of LPAs to suppress stochastic expression of HIV-1 transcripts, which may lead to sustained epigenetic silencing of integrated provirus (11, 12). Although several LPAs such as didehydro-cortistatin A (74, 75) and spironolactone (76) have displayed promising results *in vitro*, no human trials with latency enforcing compounds have been performed. In the present study, we directly compared the tCDKs CDK7, CDK9, and CDK8/19 as potential targets for enforcement of proviral latency in various cell line models as well as primary PBMCs isolated from people living with HIV-1. Our results indicate that CDK8/19 inhibitors, in particular, would contribute to the implementation of this strategy.

Recruitment of the general transcription factor TFIIH to the LTR is the rate limiting for reactivation of latent HIV-1 (19). The TFIIH complex is composed of 10 proteins consisting of a core component (XPB, XPD, p62, p52, p44, p34, p8) and a CAK module (CDK7, cyclin H, MAT1) (77). Consistent with the requirement of TFIIH for reactivation of viral transcription, we found that treatment with the selective CDK7 inhibitor YKL-5-124 prevented induction of HIV-1 in response to T cell activation (Fig. 2). Furthermore, YKL-5-124 was found to limit proviral reactivation in response to several diverse LRAs, including the TRIM24 bromodomain inhibitor IACS-9571 and the HDACi SAHA (Fig. 4). Surprisingly, inhibition of CDK7 in combination with JQ1 caused an increased proportion of GFP expressing cells in the JLat10.6 model of HIV-1 latency (Fig. 4A), although the magnitude of viral expression was severely diminished in these cells (Fig. 4B). Interestingly, we observed a similar effect when we examined reactivation of HIV-1 provirus in JLatA72 (-Tat) cells treated with YKL-5-124 (Fig. 5). These observations might also be related to our finding that inhibition of CDK7 is associated with increased susceptibility of T cells for HIV-1 infection (Fig. 7). TFIIH has both helicase and protein kinase activities that facilitate promoter opening and catalyze phosphorylation of RNAPII CTD S5 (19, 76). Our peculiar finding that inhibition of CDK7 causes an increased proportion of cells to become infected by HIV-1, albeit being enriched for latent provirus, likely represents a function of TFIIH for HIV-1 transcription that has not been characterized. We found that inhibition of HIV-1 expression by the CDK7 inhibitor YKL-5-124 only occurred at concentrations that caused significant inhibition of cell cycle progression (Fig. 6A and B), which suggests that inhibitors of CDK7 may not be appropriate for therapeutic use. The inhibitory effect of the CDK7i on cell growth likely relates to the requirement of CDK7 as the CAK, which phosphorylates and activates the cell cycle-regulated CDKs at the active site. The latency promoting agent spironolactone causes proteasomal degradation of the XBP subunit of TFIIH and was shown to enforce HIV-1 latency, though viral rebound in cells treated with this drug occurred immediately upon withdrawal (76). Furthermore, although spironolactone causes efficient reduction in XPB protein levels, it also causes reduced expression of CDK7 (76), indicating this potential LPA may also have cytotoxic effects.

The transcriptional elongation factor P-TEFb, comprised of cyclin T1 and CDK9, is recruited to the nascent HIV-1 LTR TAR RNA through interaction with Tat, and facilitates escape of promoter proximal paused RNAPII, which produces a strong positive feedback loop for HIV-1 provirus expression (78). Consistent with this defined function of CDK9, we found that a selective inhibitor of CDK9 kinase activity, LDC000067 (48), suppressed HIV-1 proviral induction in multiple HIV-1 provirus reporter cell lines (Fig. 2 and 4), and in PBMCs from people living with HIV-1 *ex vivo* (Fig. 10). We also confirmed that the function of CDK9 for HIV-1 expression is largely dependent upon the viral transactivator Tat, as LDC000067 had no appreciable effect in JLatA72 cells, which do not express Tat (Fig. 5; Fig. S5). Given the predominant role of CDK9 for HIV-1 transcription, this enzyme is an important potential therapeutic target (79, 80) and previous results have shown the CDK9 inhibitors roscovitine, flavopiridol, and seliciclib to suppress HIV-1 replication (31–33, 81). However, these previously characterized compounds are properly described as pan-CDK inhibitors as they are not selective and cause global inactivation of RNAPII transcription and cell cycle arrest (35, 36).

Importantly, in the present study, we found that the selective CDK9 inhibitor LDC000067 had no effect on cell growth or viability at concentrations effective at restricting proviral expression (Fig. 6C and D). Consequently, we were able to assess the capability of CDK9 inhibition to generate durable latency. In all T cell models examined, LDC000067 treatment generated proviral latency without affecting cell growth (Fig. 8 and 9; Fig. S7, see drug treated). However, removal of the CDK9 inhibitor from treated cells corresponded with immediate rebound of HIV-1 stochastic basal expression to comparable levels as untreated cells (Fig. 8 and 9; Fig. S7, see drug removed). There is significant interest in disrupting the Tat-CDK9 positive feedback loop for effective “block and lock” therapies, and various strategies for inhibition of Tat have been proposed, including forcing its degradation by the long noncoding RNA NRON (82) and expression of the Tat mutant designated Nullbasic (83, 84). Given that the role of Tat for HIV-1 replication involves recruitment of P-TEFb (CDK9/cyclin T1) to the 5´ LTR and that we observed that CDK9 inhibition did not generate lasting latency, our results indicate that sole disruption of the Tat positive feedback loop for expression of HIV-1 provirus is likely incapable of producing durable latency.

We have previously demonstrated that inhibition of CDK8/19 restricts HIV-1 provirus expression (41). In this report, we extend these studies to show that the CDK8/19 inhibitor Senexin A suppresses viral induction in response to a diverse set of LRAs and T cell activation (Fig. 2 and 4). Unlike CDK9, the function of CDK8/19 for HIV-1 expression is not dependent on Tat (Fig. 5; Fig. S5), consistent with previous findings that CDK8/19 inhibition limits reactivation of ACH2 and U1 proviral latency cell models (41), which have mutations that prevent activation by Tat (85, 86). We observed synergistic inhibition of HIV-1 provirus expression in cells treated with the CDK8/19 inhibitor Senexin A in combination with inhibitors of either CDK7 or CDK9, consistent with the defined role of these kinases for regulating distinct functions for RNAPII transcription. Given that Senexin A does not affect cell viability at concentrations that inhibit HIV-1 expression (Fig. 6C and D) (41), and produces a synergistic effect with the CDK9 inhibitor LDC000067, our observations indicate that uncoupling of the Tat-CDK9 positive feedback loop in combination with CDK8/19 kinase inhibition could contribute to an effective block and lock therapy. Consistent with this prediction, we observe a strong synergistic inhibitory effect of these molecules on reactivation of HIV-1 provirus in CD4 lymphocytes from infected individuals (Fig. 10, see below). CDK8/19 inhibitors seem particularly suitable for this purpose as Senexin A and BRD6989 both suppress stochastic HIV-1 reporter virus expression in multiple HIV-1 reporter cell lines, an effect that persists upon removal of the inhibitors (Fig. 8 and 9; Fig. S7 and S8). These results suggest that inhibition of CDK8/19 causes modification of transcription factors and/or chromatin organization associated with HIV-1 provirus that persists following removal of the inhibitor.

In this study, we have evaluated the potential of the transcription associated protein kinases CDK7, CDK9, and/or CDK8/19 as targets for HIV-1 antiretroviral therapies. We found that CDK7 inhibition caused cellular toxicity while also increasing the susceptibility of cell lines to HIV-1 infection. Although the CDK9 inhibitor LDC000067 was well tolerated and caused suppression of proviral expression, treatment with this inhibitor did not cause durable latency upon its removal. In contrast, we found that the CDK8/19 inhibitors Senexin A and BRD6989 restricted HIV-1 expression at concentrations that did not affect cellular growth but promoted proviral latency, an effect that was maintained for multiple days following drug withdrawal. These results indicate that inhibition of CDK8/19 causes persistent HIV-1 provirus latency, an effect that could be exploited with synergistic latency promoting agents to maintain suppression of HIV-1 provirus expression following discontinuation of ART.

## MATERIALS AND METHODS

### Cell culture, virus culture, and lentiviral transduction

Jurkat E6-1, JLat10.6, JLat9.2, JLatA72, GXR-5 CEM, Jurkat Tat mdHIV #77 and #110, and RGH #20 cell lines were cultured in Roswell Park Memorial Institute 1640 (RPMI-1640) medium supplemented with 10% fetal bovine serum (FBS), penicillin (100 units/mL), streptomycin (100 g/mL), and L-glutamine (2 mM). Human embryonic kidney cells expressing SV40 virus large T antigen (HEK293T) were cultured in Dulbecco’s modified Eagle’s medium supplemented with 10% FBS, penicillin (100 units/mL), streptomycin (100 g/mL), and L-glutamine (2 mM). All cell lines were incubated in a humidified 37°C and 5% CO_2_ atmosphere.

PBMCs from participants with HIV-1 on ART were isolated from whole blood by density gradient centrifugation using Lymphoprep and SepMate tubes (StemCell Technologies) and cryopreserved. Upon thawing, PBMCs were cultured in RPMI supplemented with 10% FBS, penicillin (100 units/mL), streptomycin (100 g/mL), and L-glutamine (2 mM).

Vesicular stomatitis virus G (VSV-G) pseudotyped viral stocks were produced by co-transfecting HEK293T cells with a combination of viral molecular clone, psPAX, and VSV-G at a ratio of 8 µg:4 µg:2 µg. Transfections were performed with polyethylenimine (PEI) at a ratio of 6:1 (PEI:DNA) in Gibco Opti-MEM. Lentiviral infections were performed by plating 1 × 10^6^ cells in 24-well plates with 500 µL RPMI containing 8 µg/mL polybrene and the amount of viral supernatant to give the desired MOI as indicated. Plates were spinoculated for 1.5 h at 1,500 rpm.

### Cell viability

Cells were stained with trypan blue solution (0.4%) and viability was determined using a Bio-Rad TC20 Automated Cell Counter.

### RT-PCR

RNA was extracted from cells using the RNeasy Kit (Qiagen) and analyzed with the Quant Studio 3 Real-Time PCR system (Applied Biosystems) using *Power* SYBR Green RNA-to-CT 1-Step Kit (Thermo Fisher) as per the manufacturer’s instructions. RT-PCR data were normalized to glyceraldehyde-3-phosphate dehydrogenase (GAPDH) expression using the ΔΔCt method as previously described (87). Cycling parameters were as follows: 48°C, 30 min, 1×; 95°C, 10 min, 1×; (95°C, 15 s, 60°C, 1 min), 60×. Primers were as follows: HIV-1 mRNA (multiply spliced Tat-Rev transcripts), Fwd 5´ CTTAGGCATCTCCTATGGCAGGA, Rev 5´ GGATCTGTCTCTGTCTCTCTCTCCACC; GAPDH, Fwd 5´ TGCACCACCAACTGCTTAGC, Rev 5´ GGCATGGACTGTGGTCATGAG.

### Flow cytometry

Cells were treated as indicated in the figure legends. Human T cell lines were suspended in phosphate buffered saline (PBS), and a BD Biosciences LSRII-561 system was used for flow cytometry with threshold forward scatter and side scatter parameters set so that a homogenous population of live cells was assessed. FlowJo software (TreeStar) was used to analyze data and determine the indicated MFI. GFP delta (Δ) MFI indicates that the MFI of the control sample has been subtracted from the results.

### Quantitative analysis of tCDK inhibitor latency promoting agent interaction

Bliss independence modeling was used as previously described (29) to statistically assess the inhibitory activity of combinatorial tCDK inhibitor treatment on HIV-1 reactivation. The equation *Fa*_xy, P_ = *Fa*_x_ *+ F*_ay_ – (*Fa*_x_) (*Fa*_y_) defines the Bliss independence model, in which *Fa*_xy, P_ is the predicted fraction affected by a combination of drug *x* and drug *y* that is derived from the experimentally observed fraction affected by drug *x* (*Fa*_x_) and drug *y* (*Fa*_y_) individually. Comparison of the predicted combinatorial affect (*Fa*_xy, P_) with the experimentally observed impact (*Fa*_xy,O_) was then performed: ∆*Fa*_xy_xy*Fa*_xy,O_ − *Fa*_xy,P_. If ∆*Fa*_xy_ is greater than 0, the combination of drugs *x* and *y* exceed that of the predicted affect indicating that the drugs display synergistic interaction. If ∆*Fa*_xy_xy0, the drug combination follows the Bliss model for independent action. If ∆*Fa*_xy_ is less than 0, the drug interaction is antagonistic as the observed effect of the drug combination is less than predicted.

### Statistics and reproducibility

All replicates are independent biological replicates, presented as mean values with ± standard deviation shown by error bars. The number of times an experiment was performed is indicated in the figure legends. LC50 and IC50 values were determined by nonlinear regression modeling using GraphPad Prism 9.0.0. *P*-values were determined using unpaired *t*-tests using GraphPad Prism 9.0.0. Statistical significance is indicated at *P < 0.05, **P < 0.01, or ***P < 0.001, with n.s. denoting non-significant *P* ≥ 0.05.

## Data Availability

All data supporting the findings of this study are available within the article or from the corresponding author upon reasonable request (I. Sadowski, ijs.ubc@gmail.com).

## SUPPLEMENTAL MATERIAL

Supplemental figures aac.01072-23-s0001.pdf Fig. S1 to S8.

ASM does not own the copyrights to Supplemental Material that may be linked to, or accessed through, an article. The authors have granted ASM a non-exclusive, world-wide license to publish the Supplemental Material files. Please contact the corresponding author directly for reuse.
