# Supplementary material for: Small molecule inhibitors of transcriptional cyclin-dependent kinases impose HIV-1 latency, presenting “block and lock” treatment strategies
Source: Antimicrob Agents Chemother. 2024 Feb 6;68(3):e01072-23. doi: 10.1128/aac.01072-23 (PMC10923280; doi:10.1128/aac.01072-23)
Supplement: Supplemental figures — Fig. S1 to S8. [file aac.01072-23-s0001.pdf]

Legends to Supplementary Figures

Figure S1. Effect of tCDK inhibition on cell viability. A – C: JLat10.6 cells were pre-treated 30 minutes with the indicated concentration of YKL-5-124 (CDK7i) (**A**), LDC000067 (CDK9i) (**B**), or Senexin A (CDK8/19i) (**C**) and were subsequently stimulated with 5 nM PMA/ 1 μM ionomycin. Following 20 hours, cell viability was assessed and the results are normalized to untreated cells (*n* = 2, mean ± SD). **D – F:** As in (**A – C**), but JLat9.2 cells were assessed (*n* = 2, mean ± SD).

Figure S2. **Effect of tCDK inhibitor combinations on cell viability. A – C:** JLat10.6 cells were pre-treated for 30 minutes with 100 nM YKL-5-124 (CDK7i) and the indicated concentration of LDC000067 (CDK9i) (**A**) or Senxexin A (CDK8/19i) (**B**), or were pre-treated 30 minutes with 1 μM LDC000067 and the indicated concentration of Senexin A (**C**). Subsequently, cells were incubated with 5 nM PMA/ 1 μM ionomycin for 20 hours. Following incubation, cell viability was determined and normalized to untreated cells (*n* = 2, mean ± SD).

Figure S3. IACS-9571 causes synergistic activation of latent HIV-1 in combination with PEP005. JLat10.6 cells were left untreated, or incubated with 5 nM PEP005, 20 μM IACS-9571, or 5 nM PEP005/ 20 μM IACS-9571. GFP expression was measured 20 hours later by flow cytometry (Fig. 4*A*). Calculation of synergy between the indicated treatments was performed using Bliss Independence Modeling. Data are presented as the difference between the

predicted and observed fractional HIV-1 expression response to the given drug combination (*n* = 2, mean ± SD). See Materials and Methods for details.

Figure S4. Effect of YKL-5-124 on background fluorescence. A: Representative flow cytometry scatter plots of wildtype Jurkat cells that were left untreated or were pre-treated for 30 minutes with 100 nM YKL-5-124 and subsequently incubated with 5 nM PMA and 1 μM ionomycin for 20 hours. **B, C:** Wildtype Jurkat cells treated as in (**A**) were analyzed by flow cytometry with GFP expression reported as the percent of GFP positive cells (**B**) and the GFP Mean Fluorescence Intensity (MFI) (**C**) (*n* = 3, mean ± SD).

Figure S5. Requirement of Tat for tCDK dependent HIV-1 expression. A: Representative flow cytometry scatter plots of GXR-5 CEM cells (-Tat) pre-treated for 30 minutes with a vehicle control (DMSO), 100 nM YKL-5-124 (CDK7i), 1 μM LDC000067 (CDK9i), or 50 μM Senexin A (CDK8/19i) and subsequently incubated with 5 nM PMA and 1 μM ionomycin for 20 hours. **B, C:** GXR-5 cells were untreated or pre-treated for 30 minutes with a vehicle control (DMSO), 100 nM YKL-5-124, 1 μM LDC00067, or 50 μM Senexin A prior to incubation with 5 nM PMA/ 1 μM ionomycin. Following 20 hours, flow cytometry was performed with viral expression reported as the percent of GFP positive cells (**B**) and the delta GFP Mean Fluorescence Intensity (MFI) (**C**) (*n* = 2, mean ± SD).

Figure S6. Effect of tCDK inhibitors on cell viability during HIV-1 infection. Jurkat human T cells were left untreated or were pre-treated for 30 minutes with 100 nM YKL-5-124, 1 μM

LDC000067, or 10 µM Senexin A. Cells were subsequently infected with RGH and 1-day post-infection, media was refreshed and cells were either washed of the drug or maintained with the same concentration of inhibitor. 4 days post-infection, viability was determined and normalized to untreated cells (*n* = 3, mean ± SD).

Figure S7. Effect of CDK9 and/or CDK8/19 inhibition on mdHIV #110 proviral latency. A: Simplified depiction of the replication incompetent mini-dual HIV-1 reporter virus that is chromosomally integrated in the mdHIV #110 Jurkat Tat clonal T cell line (51), where dsRed is expressed from the 5' LTR as a fusion with p24 Gag while an internal EF1α promoter expresses eGFP. All other viral proteins including Tat are not expressed from the mini virus. **B:** mdHIV #110 cells were incubated with a vehicle control (DMSO), 1 µM LDC000067, 10 µM Senexin A, or 1 µM LDC000067 and 10 µM Senexin A for 14 days, after which the drug was removed from the media by washing and viral expression was assessed by flow cytometry for 10 more days. **C:** Representative flow cytometry scatter plots following 4 days of drug treatment and 4 days following drug withdrawal. All "dots" are indicative of a single HIV-1 infected cell with Q2 (dsRed+/eGFP+) possessing cells with transcriptionally active provirus. **D:** mdHIV #110 cells were treated as in (**B**) with viral expression assessed on the indicated day by flow cytometry (*n* = 2, mean ± SD). **E:** mdHIV #110 cells treated as indicated in (**B**) were examined for viability, with values normalized to the vehicle control (*n* = 2, mean ± SD).

Figure S8. Effect of structurally unrelated CDK8/19 inhibitors on HIV-1 latency. A: mdHIV #77 cells were incubated with a vehicle control (DMSO), 10 µM Senexin A, or 10 µM

BRD6989 for 7 days. Subsequently, media was exchanged by washing to remove the drug and viral expression was assessed for the next 10 days by flow cytometry. **B:** Representative flow cytometry scatter plots following 4 days of drug treatment and 4 days following the removal of drug. All “dots” are indicative of a single HIV-1 infected cell with Q2 (dsRed+/eGFP+) possessing cells with transcriptionally active provirus. **C:** mdHIV #77 cells were treated as in (**A**) with viral expression assessed on the indicated day by flow cytometry (n = 2, mean ± SD). **D:** mdHIV #77 cells treated as indicated in (**A**) were examined for viability, with values normalized to cells treated with a vehicle control (n = 2, mean ± SD).

Figure S1

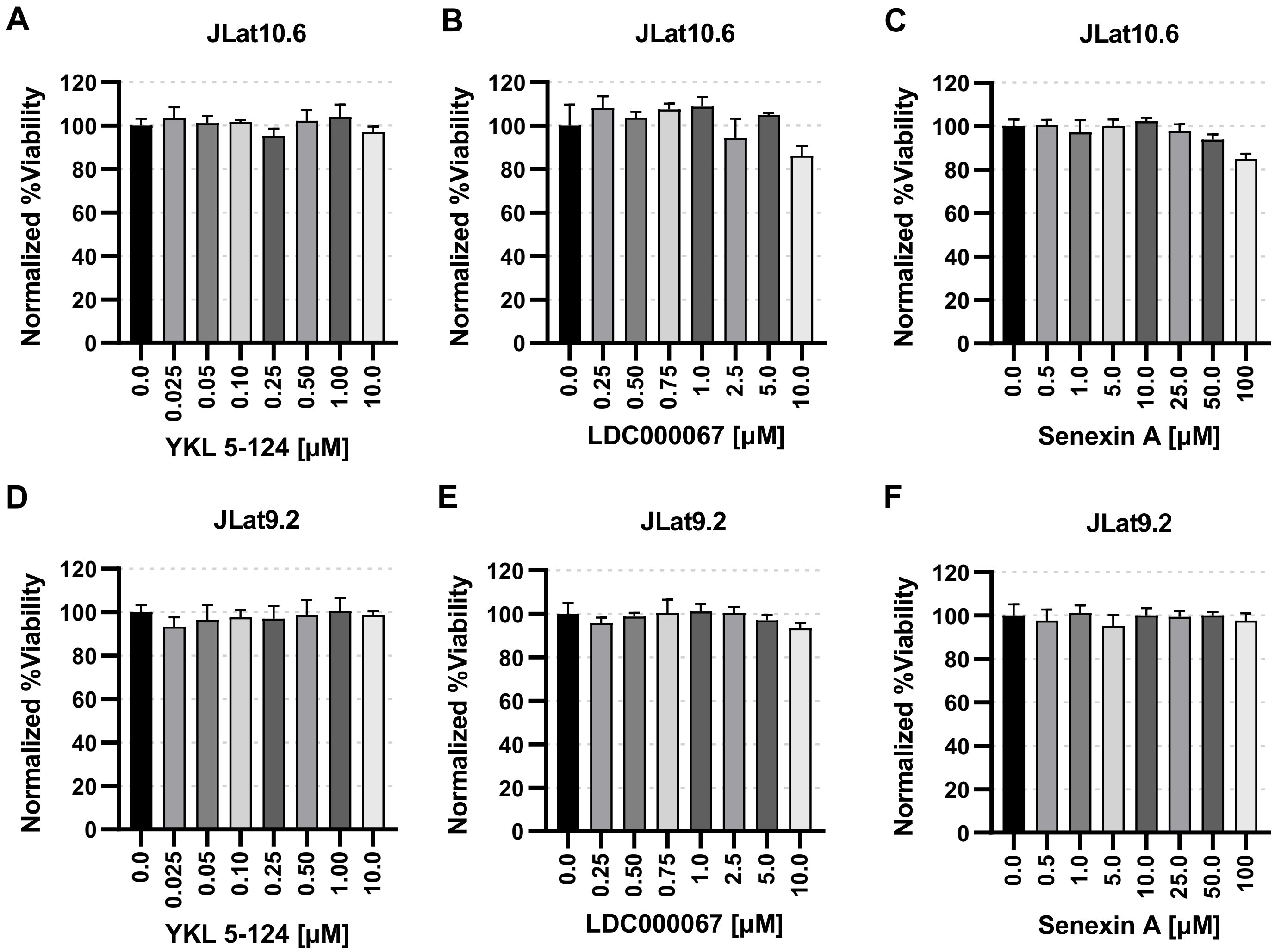

A
JLat10.6
Normalized %Viability
120
100
80
60
40
20
0
0.0
0.025
0.05
0.10
0.25
0.50
1.00
10.0
YKL 5-124 [µM]
B
JLat10.6
Normalized %Viability
0.0
0.25
0.50
0.75
1.0
2.5
5.0
10.0
LDC000067 [µM]
C
JLat10.6
Normalized %Viability
0.0
0.5
1.0
5.0
10.0
25.0
50.0
100
Senexin A [µM]
D
JLat9.2
Normalized %Viability
YKL 5-124 [µM]
E
JLat9.2
Normalized %Viability
LDC000067 [µM]
F
JLat9.2
Normalized %Viability
Senexin A [µM]

Figure S2

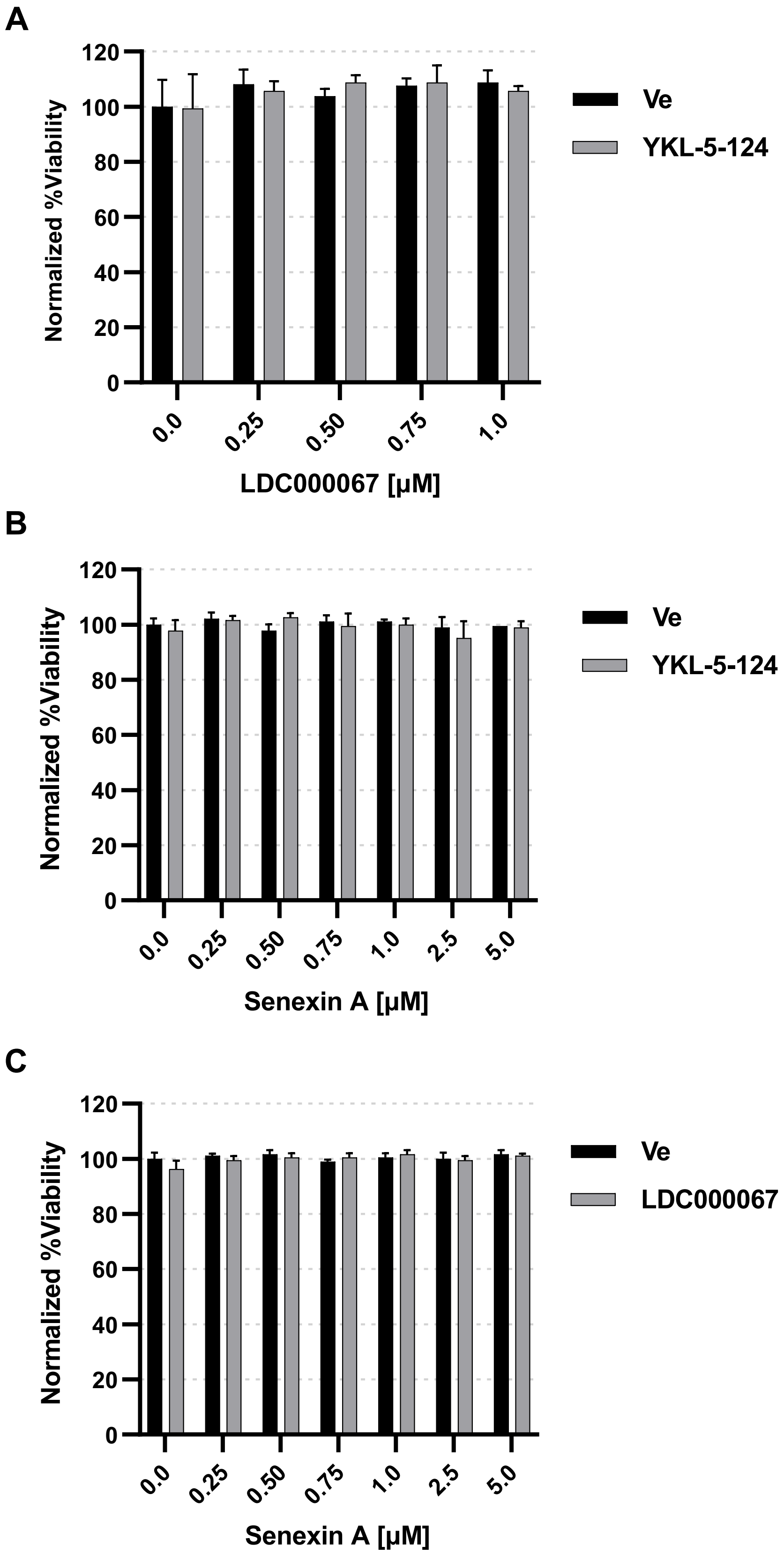

A
Normalized %Viability
120
100
80
60
40
20
0
0.0
0.25
0.50
0.75
1.0
LDC000067 [μM]
Ve
YKL-5-124
B
Normalized %Viability
120
100
80
60
40
20
0
0.0
0.25
0.50
0.75
1.0
2.5
5.0
Senexin A [μM]
Ve
YKL-5-124
C
Normalized %Viability
120
100
80
60
40
20
0
0.0
0.25
0.50
0.75
1.0
2.5
5.0
Senexin A [μM]
Ve
LDC000067

Figure S3

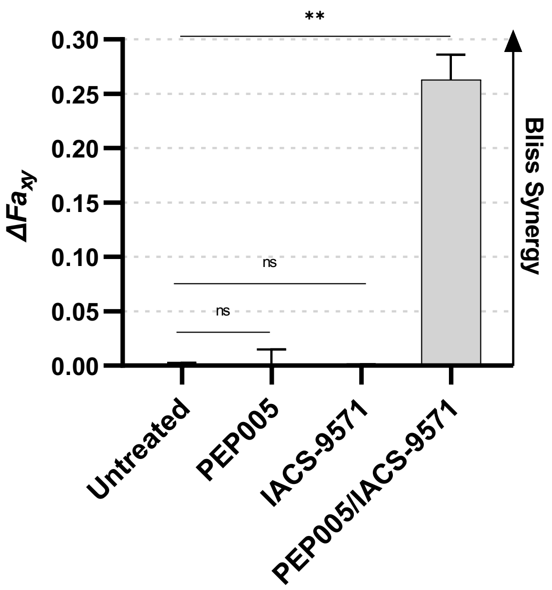

**
0.30
0.25
0.20
0.15
0.10
0.05
0.00
ΔFa$_{xy}$
ns
ns
Bliss Synergy
Untreated
PEP005
IACS-9571
PEP005/IACS-9571

Figure S4

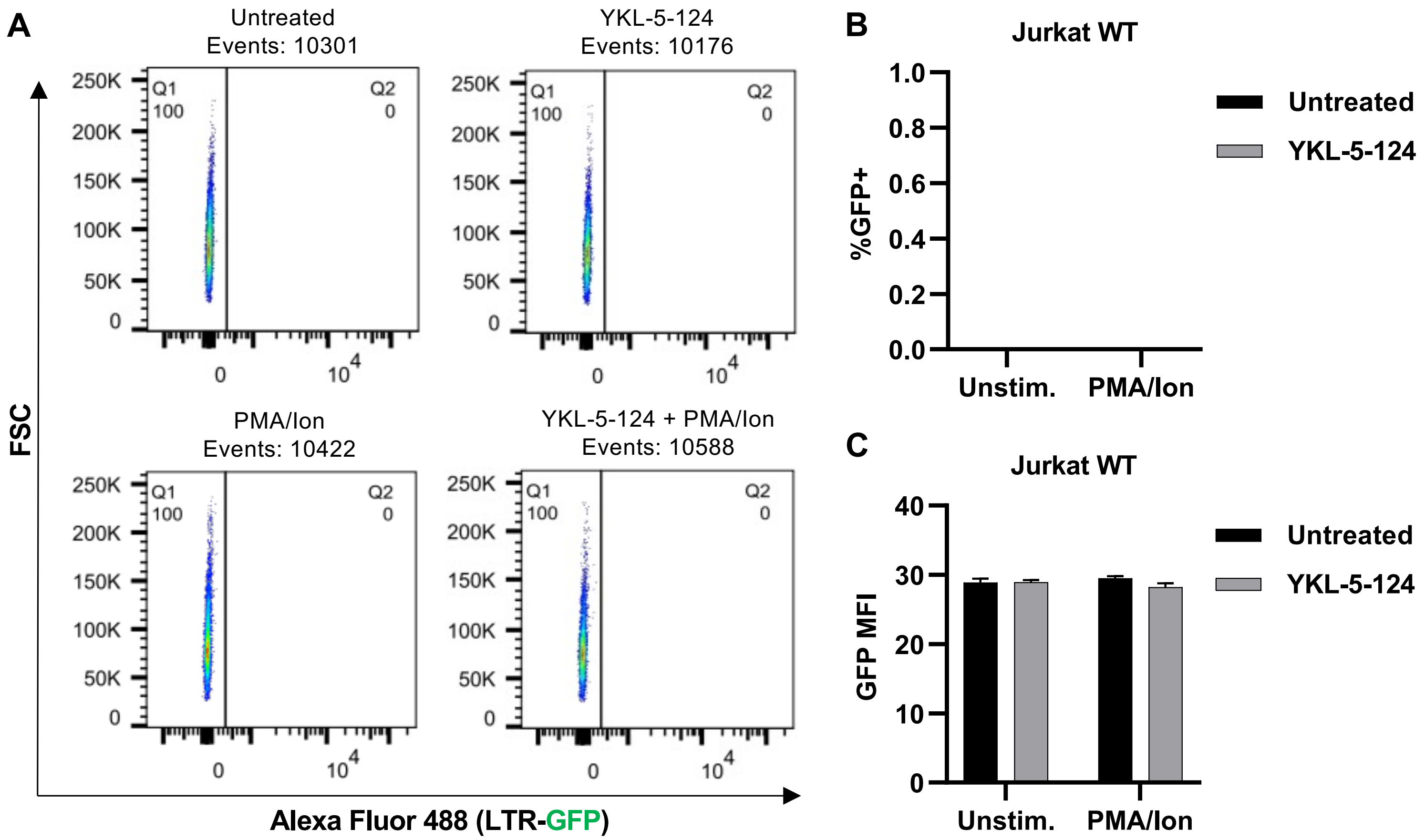
A
Untreated
Events: 10301
YKL-5-124
Events: 10176
PMA/Ion
Events: 10422
YKL-5-124 + PMA/Ion
Events: 10588
Q1
100
Q2
0
250K
200K
150K
100K
50K
0
0
10^4
FSC
Alexa Fluor 488 (LTR-GFP)
B
Jurkat WT
%GFP+
1.0
0.8
0.6
0.4
0.2
0.0
Unstim.
PMA/Ion
Untreated
YKL-5-124
C
Jurkat WT
GFP MFI
40
30
20
10
0
Unstim.
PMA/Ion
Untreated
YKL-5-124

Figure S5

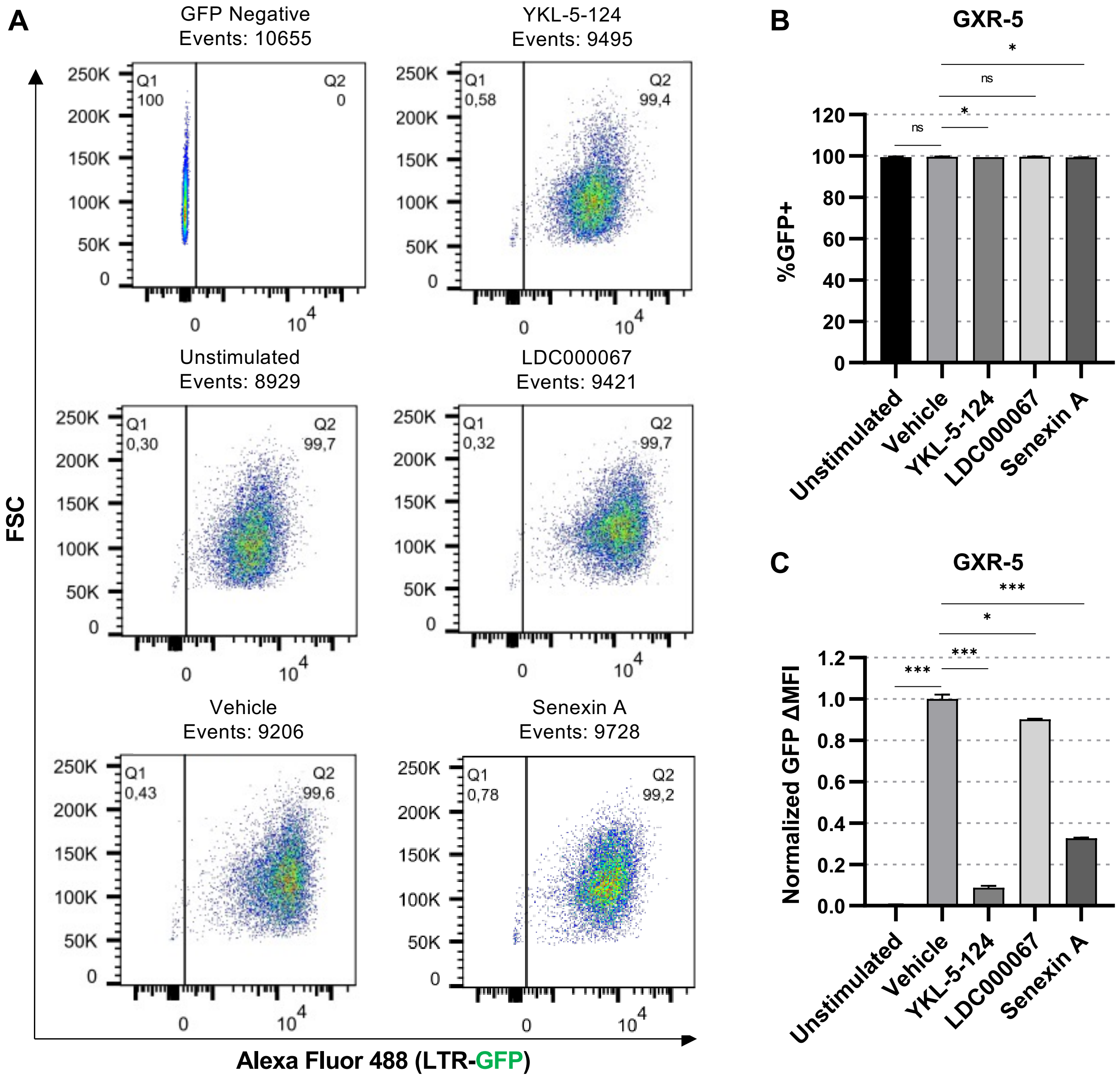

A
GFP Negative
Events: 10655
YKL-5-124
Events: 9495
Unstimulated
Events: 8929
LDC000067
Events: 9421
Vehicle
Events: 9206
Senexin A
Events: 9728
FSC
Alexa Fluor 488 (LTR-GFP)
B
GXR-5
%GFP+
C
GXR-5
Normalized GFP ΔMFI
Unstimulated
Vehicle
YKL-5-124
LDC000067
Senexin A

Figure S6

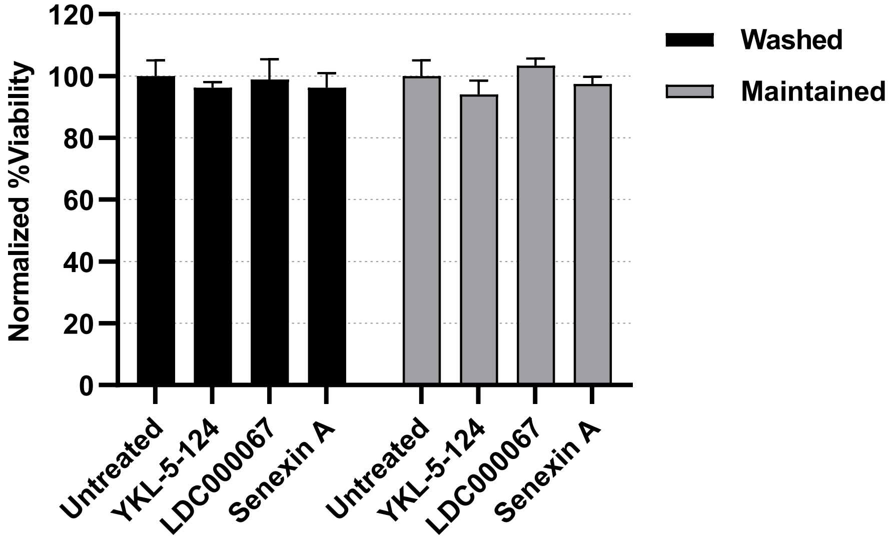

Normalized %Viability
120
100
80
60
40
20
0
Untreated
YKL-5-124
LDC000067
Senexin A
Untreated
YKL-5-124
LDC000067
Senexin A
Washed
Maintained

Figure S7

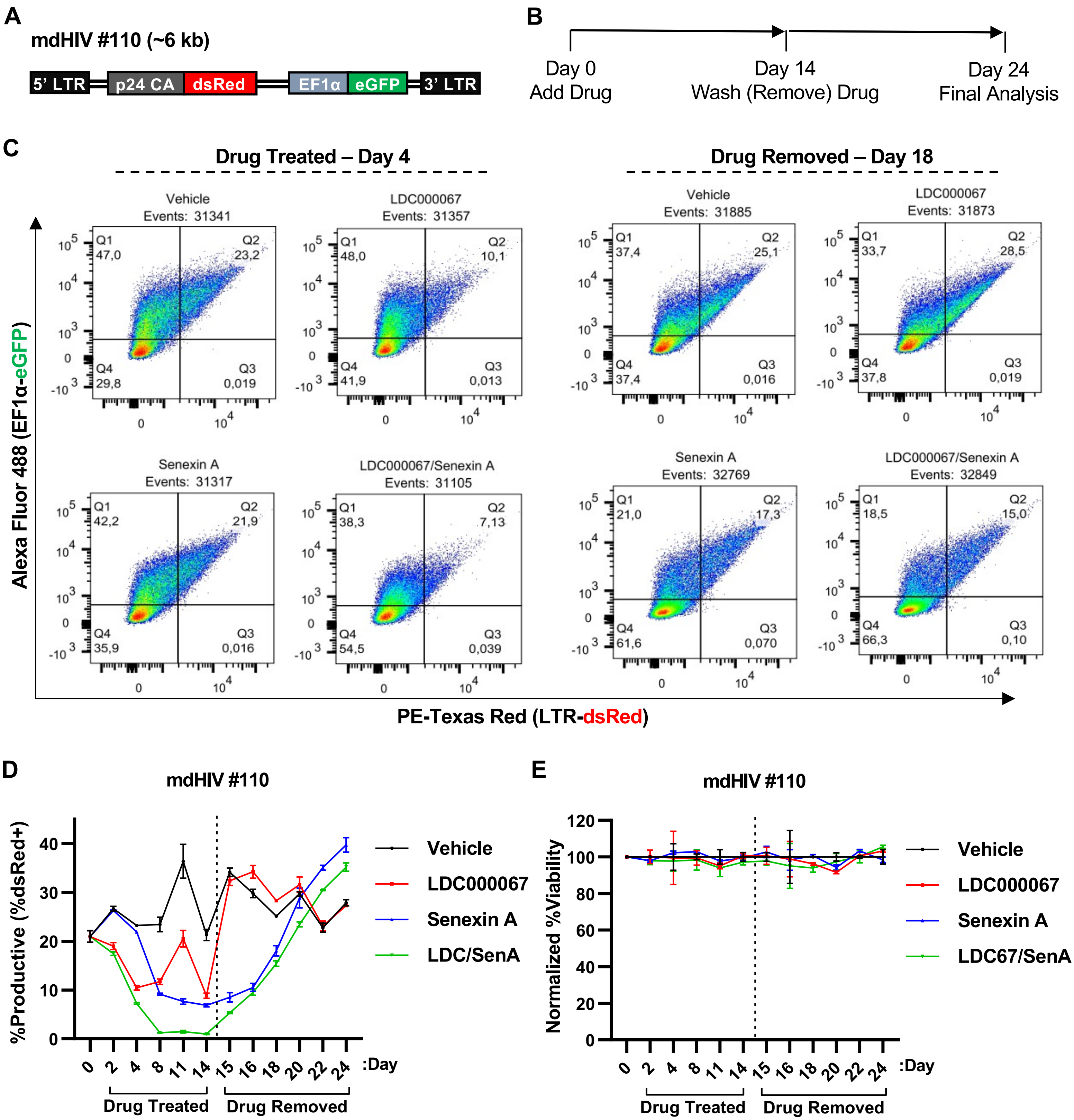
A
mdHIV #110 (~6 kb)
5' LTR
p24 CA
dsRed
EF1α
eGFP
3' LTR
B
Day 0
Add Drug
Day 14
Wash (Remove) Drug
Day 24
Final Analysis
C
Drug Treated – Day 4
Drug Removed – Day 18
Vehicle
Events: 31341
Q1 47,0
Q2 23,2
Q4 29,8
Q3 0,019
LDC000067
Events: 31357
Q1 48,0
Q2 10,1
Q4 41,9
Q3 0,013
Vehicle
Events: 31885
Q1 37,4
Q2 25,1
Q4 37,4
Q3 0,016
LDC000067
Events: 31873
Q1 33,7
Q2 28,5
Q4 37,8
Q3 0,019
Senexin A
Events: 31317
Q1 42,2
Q2 21,9
Q4 35,9
Q3 0,016
LDC000067/Senexin A
Events: 31105
Q1 38,3
Q2 7,13
Q4 54,5
Q3 0,039
Senexin A
Events: 32769
Q1 21,0
Q2 17,3
Q4 61,6
Q3 0,070
LDC000067/Senexin A
Events: 32849
Q1 18,5
Q2 15,0
Q4 66,3
Q3 0,10
Alexa Fluor 488 (EF1α-eGFP)
PE-Texas Red (LTR-dsRed)
D
mdHIV #110
%Productive (%dsRed+)
Vehicle
LDC000067
Senexin A
LDC/SenA
:Day
Drug Treated
Drug Removed
E
mdHIV #110
Normalized %Viability
Vehicle
LDC000067
Senexin A
LDC67/SenA
:Day
Drug Treated
Drug Removed

Figure S8

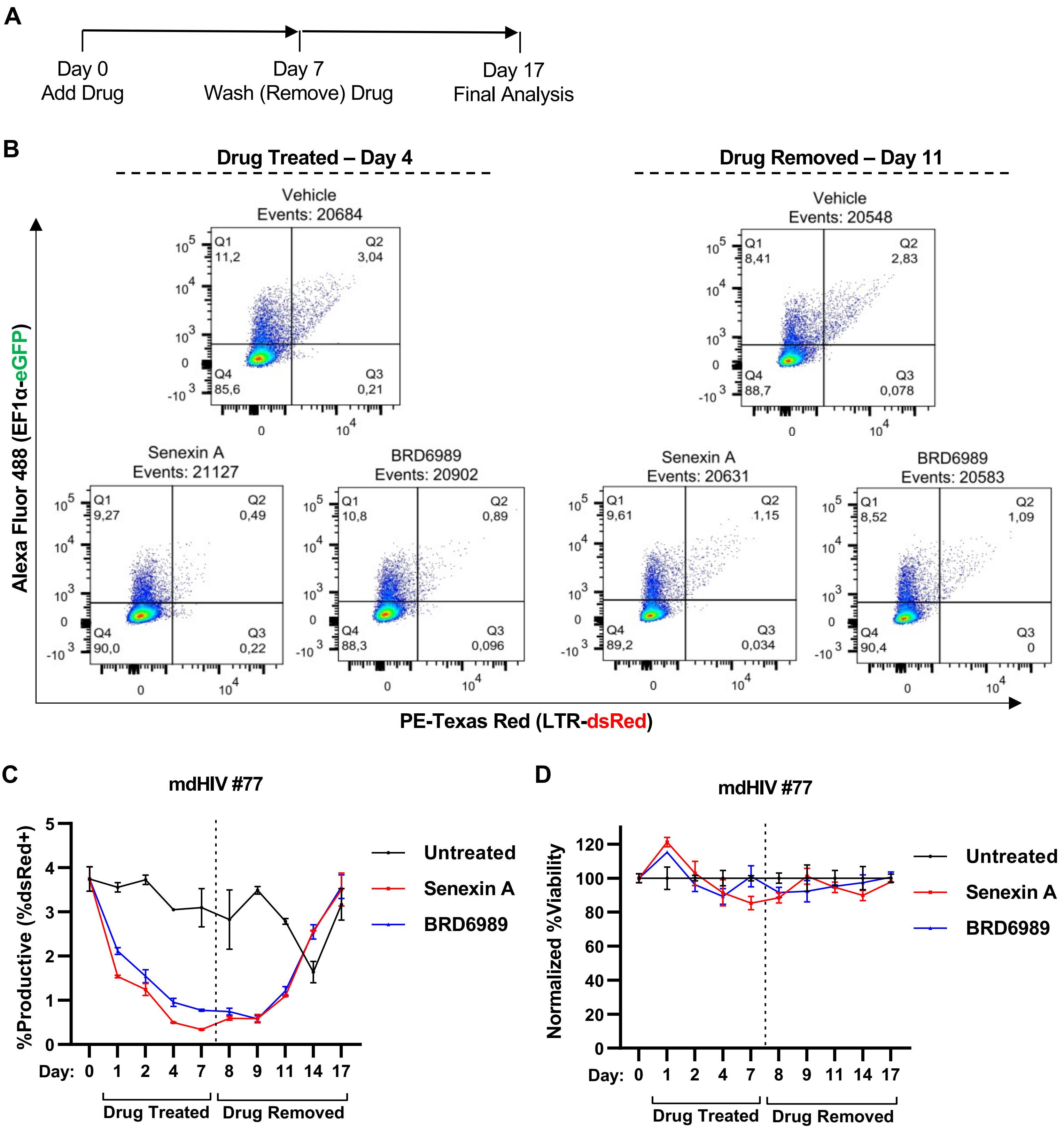
A
Day 0
Add Drug
Day 7
Wash (Remove) Drug
Day 17
Final Analysis
B
Drug Treated – Day 4
Drug Removed – Day 11
Vehicle
Events: 20684
Q1 11,2
Q2 3,04
Q4 85,6
Q3 0,21
Vehicle
Events: 20548
Q1 8,41
Q2 2,83
Q4 88,7
Q3 0,078
Senexin A
Events: 21127
Q1 9,27
Q2 0,49
Q4 90,0
Q3 0,22
BRD6989
Events: 20902
Q1 10,8
Q2 0,89
Q4 88,3
Q3 0,096
Senexin A
Events: 20631
Q1 9,61
Q2 1,15
Q4 89,2
Q3 0,034
BRD6989
Events: 20583
Q1 8,52
Q2 1,09
Q4 90,4
Q3 0
Alexa Fluor 488 (EF1α-eGFP)
PE-Texas Red (LTR-dsRed)
C
mdHIV #77
%Productive (%dsRed+)
Day: 0 1 2 4 7 8 9 11 14 17
Drug Treated
Drug Removed
Untreated
Senexin A
BRD6989
D
mdHIV #77
Normalized %Viability
Day: 0 1 2 4 7 8 9 11 14 17
Drug Treated
Drug Removed
Untreated
Senexin A
BRD6989
